# Transcriptome-based gene regulatory network analyses of differential cold tolerance of two tobacco cultivars

**DOI:** 10.1186/s12870-022-03767-7

**Published:** 2022-07-26

**Authors:** Zhenyu Luo, Zhicheng Zhou, Yangyang Li, Shentong Tao, Zheng-Rong Hu, Jia-Shuo Yang, Xuejiao Cheng, Risheng Hu, Wenli Zhang

**Affiliations:** 1 State Key Laboratory for Crop Genetics and Germplasm Enhancement, CIC-MCP, Nanjing Agricultural University, No.1 Weigang, Nanjing, 210095 Jiangsu China; 2 Hunan Tobacco Research Institute, Changsha, 410128 Hunan China

**Keywords:** Transcriptome, Differential gene expression, Co-expression network, Cold treatment, Tobacco

## Abstract

**Background:**

Cold is one of the main abiotic stresses that severely affect plant growth and development, and crop productivity as well. Transcriptional changes during cold stress have already been intensively studied in various plant species. However, the gene networks involved in the regulation of differential cold tolerance between tobacco varieties with contrasting cold resistance are quite limited.

**Results:**

Here, we conducted multiple time-point transcriptomic analyses using Tai tobacco (TT, cold susceptibility) and Yan tobacco (YT, cold resistance) with contrasting cold responses. We identified similar DEGs in both cultivars after comparing with the corresponding control (without cold treatment), which were mainly involved in response to abiotic stimuli, metabolic processes, kinase activities. Through comparison of the two cultivars at each time point, in contrast to TT, YT had higher expression levels of the genes responsible for environmental stresses. By applying Weighted Gene Co-Expression Network Analysis (WGCNA), we identified two main modules: the pink module was similar while the brown module was distinct between the two cultivars. Moreover, we obtained 100 hub genes, including 11 important transcription factors (TFs) potentially involved in cold stress, 3 key TFs in the brown module and 8 key TFs in the pink module. More importantly, according to the genetic regulatory networks (GRNs) between TFs and other genes or TFs by using GENIE3, we identified 3 TFs (ABI3/VP1, ARR-B and WRKY) mainly functioning in differential cold responses between two cultivars, and 3 key TFs (GRAS, AP2-EREBP and C2H2) primarily involved in cold responses.

**Conclusion:**

Collectively, our study provides valuable resources for transcriptome- based gene network studies of cold responses in tobacco. It helps to reveal how key cold responsive TFs or other genes are regulated through network. It also helps to identify the potential key cold responsive genes for the genetic manipulation of tobacco cultivars with enhanced cold tolerance in the future.

**Supplementary Information:**

The online version contains supplementary material available at 10.1186/s12870-022-03767-7.

## Background

Sessile plants are constantly subjected to various abiotic clues during the lifecycle such as drought, salinity, heat and cold. These stresses dramatically limit their normal growth and development, and final productivity as well [1]. Naturally occurring cold stress usually causes complex symptoms including growth retardation, leaf chlorosis, wilt and droop, chilling/frost/freeze injury and final death of tissues or plants [2–4]. Therefore, cold stress is considered as one of the most destructive threats that adversely affect plant lifecycle [5, 6]. Plants have to adjust multilayer transient changes, including physiological, biochemical, metabolic and molecular alterations, to adapt cold stress [4, 7–12].

Gene expression change is one of adaptive molecular mechanisms for plants responding to cold stress. In particular, rapid up-regulation of cold-responsive genes, such as C-REPEAT BINDING FACTOR (CBF) genes, COR (Cold Regulated) genes, and other key regulons can enhance cold tolerance [7, 12–18]. With the advent of high throughput sequencing, transcriptomic studies have been widely applied in many plant species, including model plants [19], crops, horticulture plants [20–23], vegetables [24–28], trees [29, 30] and tobacco [31, 32]. These studies provide insights into how transcriptional changes occur during cold stress.

Tobacco (*Nicotiana tabacum*), originally, is a tropical and subtropical plant. It currently becomes commercially popular economic crops grown worldwide. Moreover, tobacco leaves and BY-2 cells provide an excellent system for studies of functional genomics and metabolism [33, 34]. However, tobacco is overall highly susceptible to cold stress [35–38]. Mechanisms underlying cold responses in tobacco have already been investigated at physiological, biochemical, transcriptional, and metabolic levels. Cold acclimation helps tobacco to cope with cold stress through improving antioxidant enzymatic activities, osmotic adjustment potentials and photochemical efficiency [39], these changes are partially caused by reprogramming gene expression and metabolites [31]. Cold treatment can change accumulation levels of certain functional proteins involved in photosynthesis, protein/RNA procession and redox etc. [40]. *NtbHLH123* acts as a positive regulator to activate expression of cold responsive genes, *NtCBF* (C-repeat binding factor) and alleviate damage of ROS (reactive oxygen species), thereby enhancing cold resistance [41]. Moreover, functional studies showed that tobacco plants with overexpression of chloroplast ω − 3 fatty acid desaturase gene [36], *TaWRKY10* [42], *Capsella bursa-pastoris CE53* and *CBF genes* (*CblCE53* and *CbCBF*) [43] exhibit enhanced cold tolerance, indicating that these genes play positive roles in tobacco cold responses. Thus, elucidating the underlying mechanisms responsible for cold responses, especially identifying key cold-responsive genes, can facilitate the development of genetically modified tobacco varieties with cold tolerance. However, the gene networks related to cold responses are still understudied in tobacco.

## Results

### Identification of differentially expressed genes between two cultivars under cold treatment

Yan tobacco (YT) and Tai tobacco (TT) are two important tobacco cultivars for tobacco production, but exhibit contrasting cold responses. YT is cold resistant whereas TT is susceptible to cold stress. To investigate transcriptional changes between two cultivars under cold stress, we conducted RNA-seq with 6 time points before (0 h, CK) and after cold treatment at 4 °C (0.5 h, 1 h, 2 h, 4 h and 8 h), three biological replicates per sample (Additional file 2: Table S1). Correlation analyses showed that replicated data were generally well correlated within the same time point in YT (Additional file 1: Fig. S1A) or TT (Additional file 1: Fig. S1B).

**Fig. 1:**
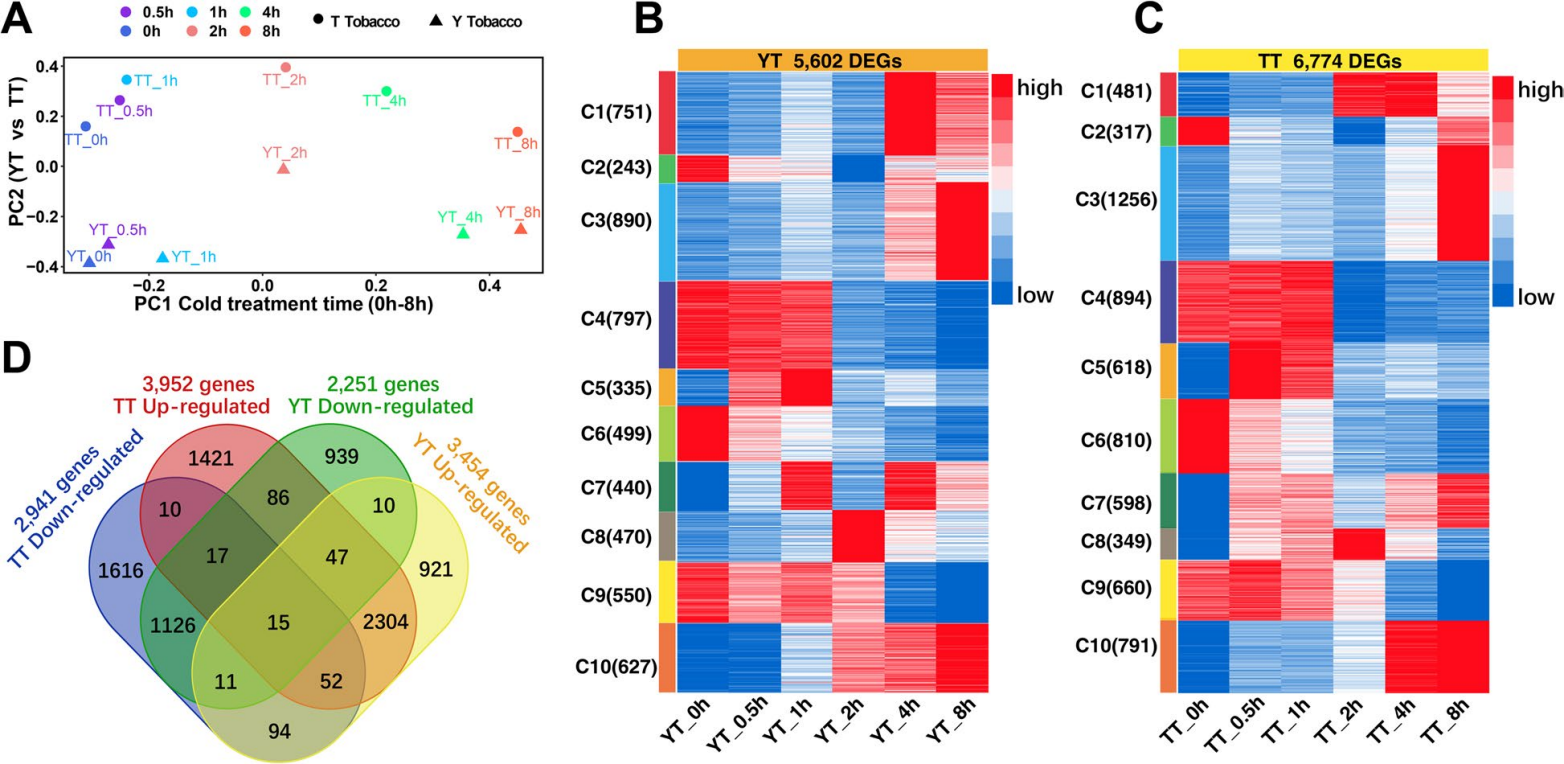
Characterization of differentially expressed genes before and after cold treatment in TT or YT. **A** Principal Component Analysis (PCA) of gene expression profiles in TT and YT before and after cold treatment. Each point represents one treatment for gene expression profiling. **B**, **C** Heatmaps showing genes differentially expressed at each time point in YT (**B**) or TT (**C**) before (0 h) and after cold treatment (0.5, 1, 2, 4, 8 h). 5602 DEGs in YT and 6774 DEGs in TT were analyzed by using k-means clustering. Each heatmap was generated through k-means clustering algorithm with the Z-score result of gene expression in Cluster 3.0 software. The color key representing the standardized gene expression levels from high (red) to low (blue). **D** Venn plots showing comparisons of genes that were up-regulated or down-regulated after cold treatment between YT and TT

To examine time-point-related transcriptional changes in YT and TT after cold treatment, we conducted a Principal Component Analysis (PCA), and observed that PC2 was quite different between TT and YT (Fig. 1A). YT and TT had a similar trend of time-point-related expression changes after cold treatment, slight expression changes occurred before 1 h, whereas dramatic expression changes occurred after 2 h (Fig. 1A). We then identified differentially expressed genes (DEGs) occurred in TY and TT at each time point after exposure to cold treatment compared with CK (Additional file 2: Table S2). We found that TT had more DGEs, especially for down-regulated genes, than YT at the same time point, suggesting that more genes are dynamic in TT relative to YT during cold stress. To assess cold-induced transcriptional changes in YT or TT, we counted total cold-induced DEGs, one of which differentially expressed at least at one time point, in YT (5622 DEGs) and TT (6799 DEGs). We then standardized the expression of DEGs by removing some extreme expression values and generated a heatmap with 10 subclusters to visualize time point related changes in YT (5602 DEGs, Fig. 1B) or TT (6774 DEGs, Fig. 1C) and between YT and TT. Time point related similar and distinct changes were observed between YT and TT in response to cold stimulus. After conducting pair-wise comparisons, we found that 1169 and 2418 genes were down- and up-regulated in TT and YT, 337 genes (165 genes up-regulated in TT but down-regulated in YT, and 172 genes down-regulated in TT but up-regulated in YT) exhibited opposing expression changes between TT and TY, and the rest of up- or down-regulated genes were specific to TT or YT (Fig. 1D).

To assess if DEGs are related to any significant biological relevance, we conducted GO term enrichment analyses using up- and down-regulated genes. We found that up-regulated genes had much more GO terms than down-regulated genes (Additional file 1: Fig. S2). Moreover, we found that GO terms differentially occurred in up-regulated genes specific to TT or YT. GO term functions in signal/molecular transduce activity, responses to stimuli, receptor/binding activity and (cellular) protein/primary metabolic processes were significantly enriched in the up-regulated genes in TT instead of YT. In contrast, functions in thylakoid, photosynthesis, membrane, extracellular region and generation of precursor metabolites and energy were overrepresented in the up-regulated genes in YT but not in TT (Additional file 1: Fig. S2). Furthermore, GO terms differentially occurred in genes up-regulated in TT but down-regulated in YT and genes down-regulated in TT but up-regulated in YT (Additional file 1: Fig. S2).

**Fig. 2:**
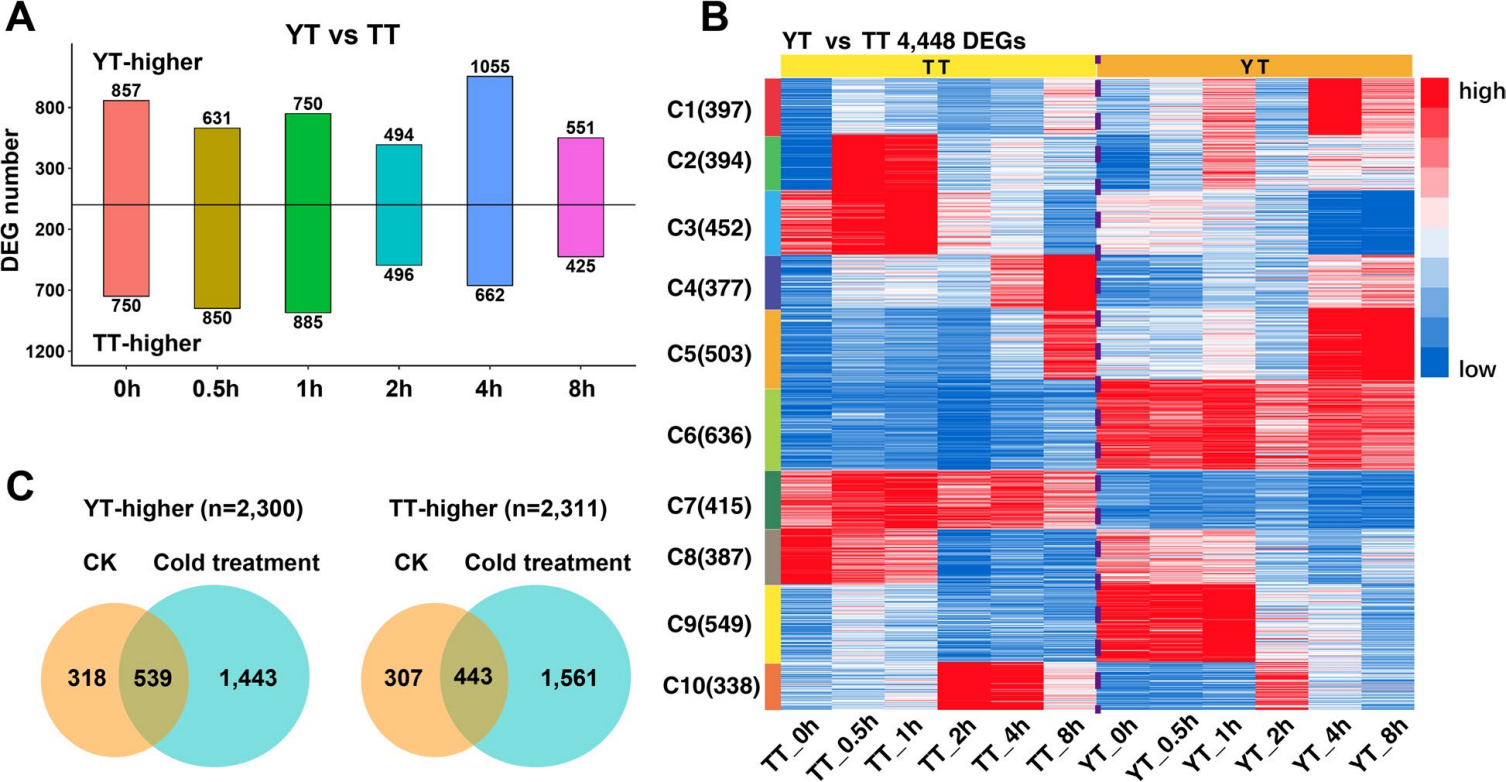
Characterization of differentially expressed genes between YT and TT in each time point. **A** Box plots showing the number of DEGs (TY or TT-higher) occurred at each time point. **B** Heatmap showing expression profiles of DEGs at each time point for YT vs TT. 4448 DEGs were analyzed by hierarchical clustering. The heatmap was generated through k-means clustering algorithm with the row-wise Z-score result of gene expression in Cluster 3.0 software. 4448 DEGs were grouped into 10 distinct subclusters according to gene expression profiles. The color key representing the standardized gene expression levels from high (red) to low (blue). **C** Venn plots showing comparison of genes that were up-regulated or down-regulated before (CK) or after cold treatment

To further assess cold-induced transcriptional changes between YT and TT, we identified DEGs occurred between YT and TT at each time point (Fig. 2A). We noticed that there were 857 YT-higher genes and 750 TT-higher genes before cold treatment (CK), reflecting genotype-related gene expression changes. As shown in Fig. 2B, 4,448 DEGs between YT and TT were grouped into 10 subclusters for showing genotype-related gene expression changes at each time point. For example, DEGs in C6 and C7 were highly expressed in YT and TT, respectively, at almost each time point. To assess if the DEGs in each cluster have any significantly functional enrichment, we conducted GO term enrichment analyses. We observed variations in GO terms among 10 subclusters (Additional file 1: Fig. S3). DEGs in C10 exhibited highly enriched GO functions related to metabolic processes, membrane and thylakoid; GO functions of DEGs in C6 and C9 were overrepresented in response to stresses. Taken together, all above analyses indicate DEGs display variations in the expression profiles and GO terms functions at each time point between TT and YT, thereby resulting in changes in response to cold treatment between TT and YT.

Compared with CK, we identified 1443 YT-higher and 1561 TT-higher genes under cold treatment (Fig. 2C). According to GO term enrichment analyses, we observed distinct GO terms associated with up- or down-regulated genes in CK and cold treatment (Additional file 1: Fig. S4). In general, up-regulated genes in CK were mainly responsible for some fundamental biological functions such as DNA/protein/nucleotide binding activities and kinase/hydrolase/pyrophosphatase activities, whereas cold-induced up- or down-regulated genes were mainly enriched in some biologically regulatory functions, responses to stresses, photosynthesis-related functions and reproduction. Collectively, transcriptomic analyses show that TT and YT have some specific DEGs functioning in distinct biological processes during cold treatment, which possibly cause differential cold responses between YT and TT.

### Identification of differentially expressed TFs and protein kinases under cold treatment

It has been documented that transcription factors (TFs) [44, 45] and various types of protein kinases (PKs) [46–48] act as key regulators in response to abiotic/biotic stresses in plants. To assess how TFs and PKs are differentially expressed in TT/YT or between TT and YT under cold treatment, we classified all DEGs in TT/YT or between TT and YT into TFs, PKs and other genes (Additional file 1: Fig. S5). Compared with TT, YT had a similar number of down-regulated TFs and PKs but have less up-regulated TFs and PKs. Moreover, we observed variations in percentage of down- and up-regulated TFs in TT/YT or between TT and YT (Additional file 1: Fig. S6A). For instance, WRKY, MYB, NAC and AP2-EREBP TFs had relatively higher percentage than other up-regulated TFs in TT and YT. GRAS, C2H2 and bHLH TFs had similar percentage in both down- and up-regulated TFs in TT and YT. Similarly, we also observed variations in percentage of down- and up-regulated PKs in TT/YT or between TT and YT (Additional file 1: Fig. S6B). More Leucine Rich Repeat (LRR) Kinases XI&XII were down-regulated than other PKs in TT and YT, while more LRR Kinase VII and III were down-regulated in TT relative to YT.

**Fig. 3:**
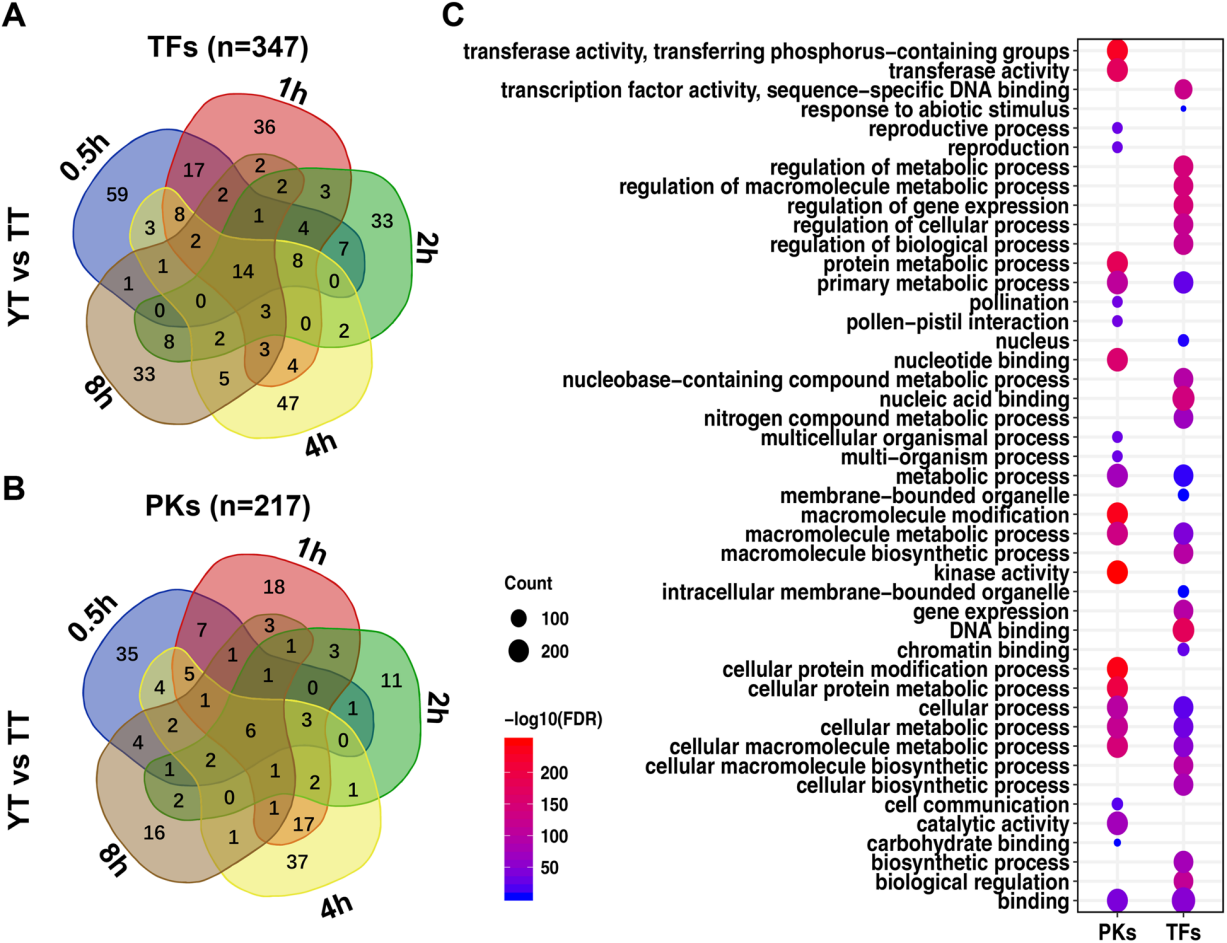
TFs or PKs differentially expressed between YT and TT under cold treatment. **A**, **B** Venn plots showing TFs (**A**) or PKs (**B**) differentially expressed at each time point between YT and TT after cold treatment (0.5 h, 1 h, 2 h, 4 h, 8 h). **C** GO term enrichment analyses of TFs or PKs that differentially expressed at least at one time point between YT and TT before (0 h) and after cold treatment (0.5 h, 1 h, 2 h, 4 h, 8 h)

To examine time-point-dependent expression changes of TFs and PKs under cold treatment, we conducted pairwise comparisons using differentially expressed TFs and PKs in TT and YT (Additional file 1: Fig. S7). For 609 TFs/384 PKs in TT (Additional file 1: Fig. S7A, B) and 526 TFs/303 PKs in YT (Additional file 1: Fig. S7C, D), which were differentially expressed at least at one time point after cold treatment, 89 TFs/79 PKs in TT and 47 TFs/19 PKs in YT were differentially expressed across all time points (Additional file 2: Table S3), other TFs and PKs were differentially expressed at one time point under cold treatment. Similarly, we conducted similar analyses using 347 TFs (Fig. 3A) and 217 PKs (Fig. 3B) differentially expressed at least at one time point between TT and YT under cold treatment. Compared with TT, we found that 14 TFs/6PKs were differentially expressed across all time points under cold treatment (Additional file 2: Table S3).

After conducting GO term enrichment analyses, we found that 347 PKs had significantly enriched GO terms with functions in transferase activities, (cellular) protein metabolic processes, macromolecular/cellular protein modifications, kinase activities; as expected, GO terms with functions of 217 TFs were mainly overrepresented in transcription factor activities, nucleic acid/DNA binding, regulation of gene expression and metabolic processes and biological regulations (Fig. 3C). Based on the above analyses, we found that TFs differentially expressed in the two samples after cold treatment mainly included AP2-EREBP, BHLH, WRKY, MYB TF family, and protein kinases mainly included Leucine Rich repeat, Plant External Response Like Kinase and Calcium Dependent Protein Kinase. In addition to some of TFs or protein kinases already reported in cold responses of different plant species [41, 49], we found that some new genes such as *Nitab4.5_0002105g0060* (*C2H2*) (Additional file 2: Table S3) were potentially in relation to cold responses, which are necessary to be functionally validated in tobacco or other plants.

### Weighted gene co-expression network analysis (WGCNA)

**Fig. 4:**
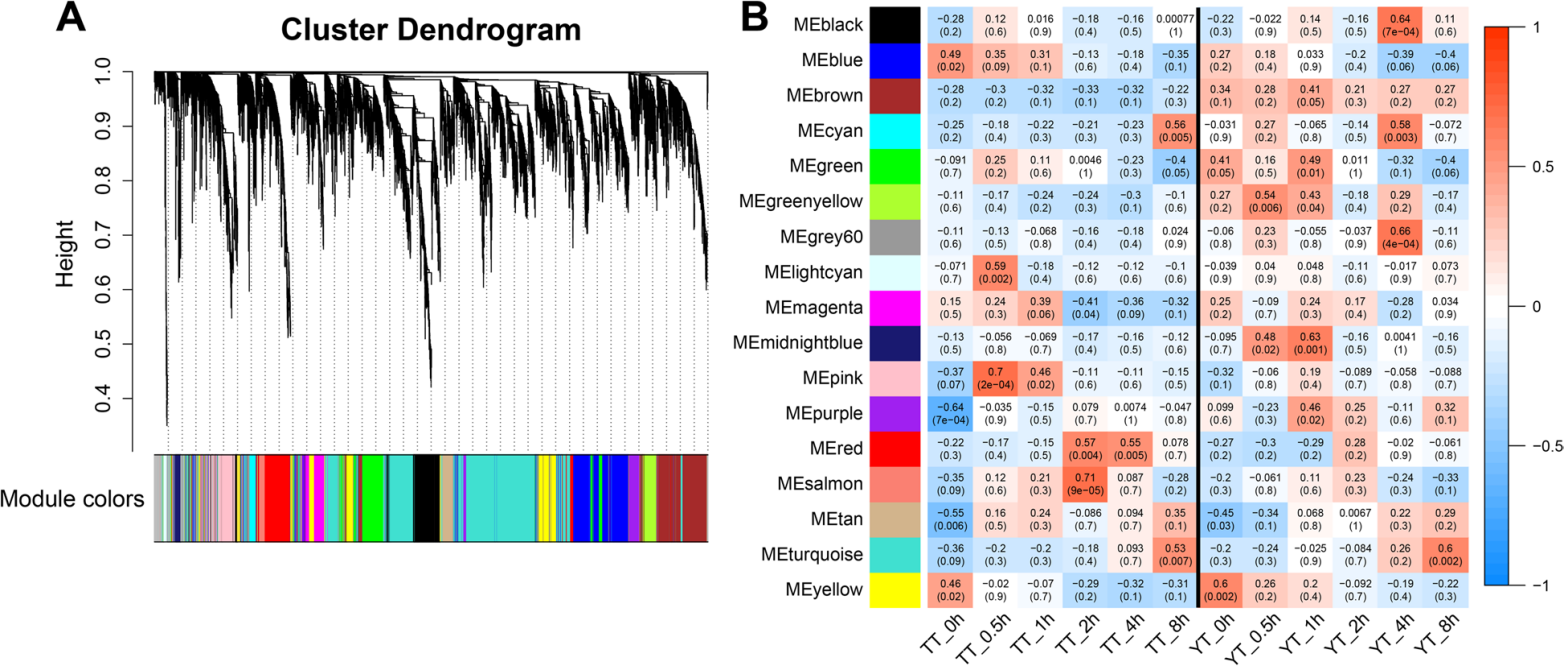
Identification of co-expression gene modules using differentially expressed genes. **A** Hierarchical cluster tree showing co-expression modules identified by WGCNA. Each leaf in the tree represents one gene. The major tree branches constitute 17 modules labelled by different colors. The *x*-axis represents genes, and the *y*-axis represents the co-expression distance. Modules were identified using dynamic tree cutting by dividing the dendrogram at significant branch points. The horizontal bar immediately below the dendrogram represents modules indicated with different colors. **B** Module-time point correlation. Each row represents a module. Each column corresponds to a specific sample. The color of each cell at the row–column intersection indicates the correlation coefficient between the module and sample. Red indicates a high degree of correlation between a specific module and the sample

To further unveil if hub genes or TFs are involved in cold responses through interacting with other genes or TFs in TT and YT, we conducted Weighted Gene Co-Expression Network Analysis (WGCNA) [50] using 9583 DEGs that were differentially expressed at least at one time point in TT or YT after cold treatment. In particular, WGCNA specifically focused on two well correlated biological replicates (Additional file 1: Fig. S8). We obtained 17 co-expression modules visualized using different colors according to the WGCNA package function (Fig. 4A). After extracting the number of genes and TFs in each module, we observed variations in the number of genes or TFs per module, ranging from 38 genes and 2 TFs in the grey60 module to 2258 genes and 233 TFs in the turquoise module with an averaged number as 488 genes and 44 TFs (Additional file 1: Fig. S9). In addition, we constructed module-time point correlation heatmap to clearly visualize the correlation between each module and each time point (Fig. 4B). We also examined the overall expression trend of co-expressed genes in each module at each time point (Additional file 1: Fig. S10). We found that the overall expression levels of genes in the brown module at each time was higher in YT than in TT; Genes in the pink module tended to be up-regulated at the first time point of cold treatment (0.5 h) in YT and TT.

### Identification of key genes involved in cold responses in tobacco

**Fig. 5:**
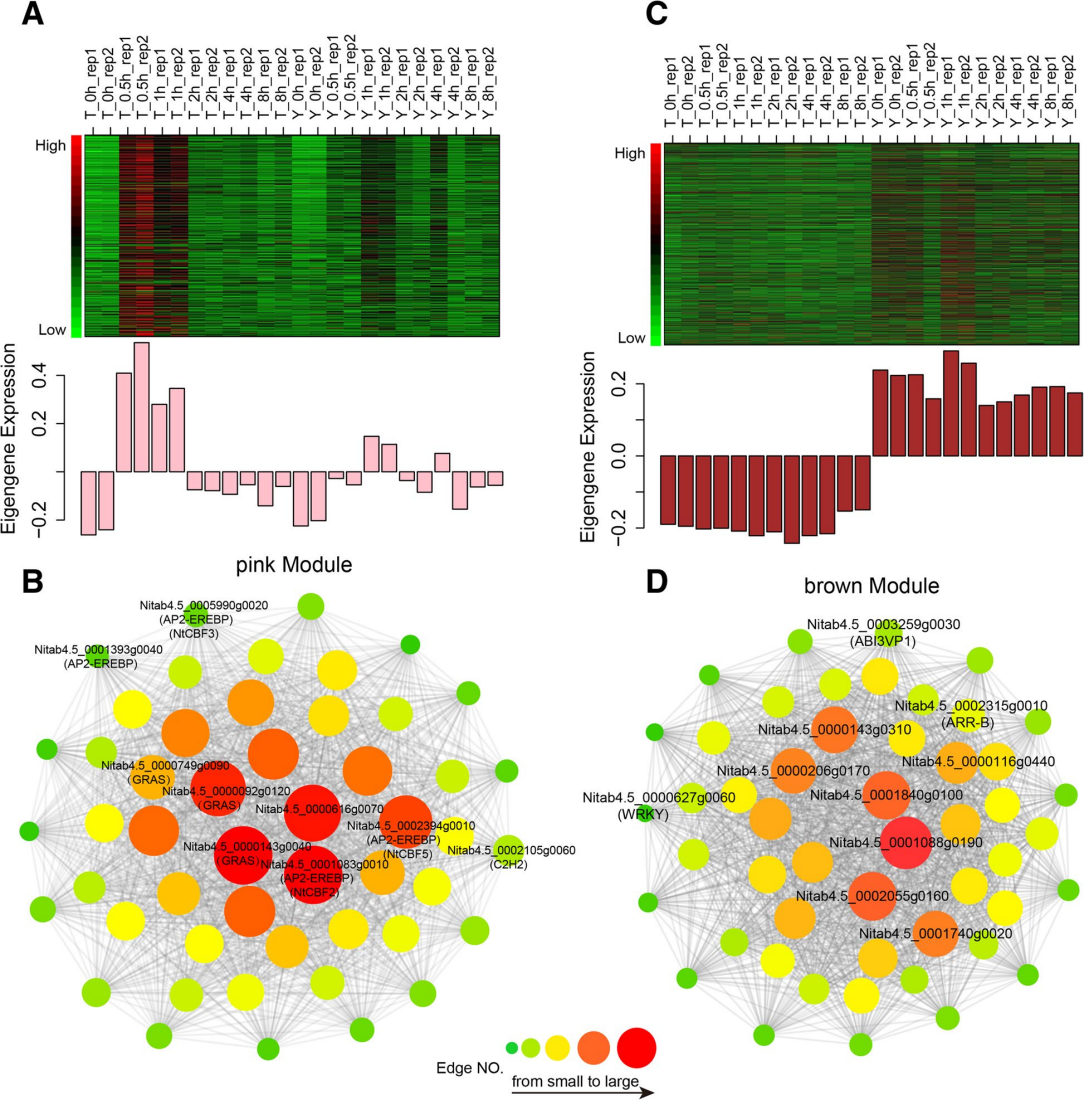
Analyses of the pink and brown module. **A** Module gene expression pattern of the brown module, red indicates high expression level and green indicates low expression level. **B** The correlation network of the pink module. A gene network is constructed by using WGCNA, in which each node represents a gene; the connecting line (edge) between genes represents the co-expression correlation. The genes with edge weights > 0.1 and sorting the top 50 by the number of edges are visualized by Cytoscape. The size and color of each circle represent the number of edges. **C** Module gene expression pattern of the brown module, red indicates high expression level and green indicates low expression level. **D** The correlation network of the pink module. A gene network is constructed by using WGCNA, in which each node represents a gene; the connecting line (edge) between genes represents the co-expression correlation. The genes with edge weights > 0.1 and sorting the top 50 by the number of edges are visualized by Cytoscape. The size and color of each circle represent the number of edges

We connected the 17 modules with each time point post cold treatment in TT and YT. According to module-time point association, we found that the correlation coefficient between genes in the pink module and time points was very low before cold treatment (0 h), while became very high at 0.5 h after cold treatment in TT. A similar trend occurred in YT, but the change was relatively weak (Figs. 4B and 5A). Meanwhile, the pink module containing 471 genes, including 80 TFs, was the module with the most TF relative to the other modules (Additional file 1: Fig. S9). To assess roles of genes in the pink module in cold responses, we conducted GO term enrichment analyses, and observed that they were mainly involved in various biological and metabolic processes (Additional file 1: Fig. S11A). GO terms related to the regulation of gene expression, metabolic processes, cellular processes and biological processes and DNA binding were enriched for genes after cold treatment, which is consistent with the possibility that plants adjust changes responding to cold treatment.

To further assess if genes in the pink module are involved in cold responses through interacting with other genes. We generated a co-expression network containing genes with edge weights > 0.1 and the top 50 number of edges, which were derived from DEGs in the pink module. We obtained the top 50 hub genes in the pink module, which contain 8 TFs and 42 other genes (Fig. 5B, Additional file 2: Table S4). Specific descriptions of hub genes in the network are listed in Additional file 2: Table S4. We indeed found some important cold-related and stress-related genes, such as *NtCBF* [41] that play important regulatory roles in coping with cold stress [18], other AP2-EREBP, GRAS and C2H2 TF family.

### Identification of genes responsible for differential cold responses between TT and YT

After a closer examination, we found that only the brown module exhibited a high association with all time-points in YT instead of TT (Fig. 4B), suggesting that this module may contain genes responsible for cold tolerance in YT. Consistently, we found that genes in the brown module were mainly up-regulated at all time-points in YT as compared to TT (Fig. 5C). To assess if co-expression genes in the brown module are possibly involved in differential cold responses between TT and YT, we performed GO term enrichment analyses, and found that they were mainly involved in responses to stress or stimuli (Additional file 1: Fig. S11B).

Similarly, we conducted a co-expression network containing genes with edge weights > 0.1 and the top 50 number of edges, which were derived from DEGs in the brown module (Fig. 5D). We found that the co-expression network contained some hub genes like *RAP74*, UDP-Glycosyltransferase, *WRKY21*, *ABI3/VP1* (ABSCISIC ACID INSENSITIVE 3/VIVIPAROUS 1, RAV), *PSP* (3-phosphoserine phosphatase) and *ARR12* (type A response regulator 12) genes (Additional file 2: Table S5). Some of these genes have been found to be involved in stress responses or signal transduction in plants [51–56]. Moreover, *AtRAP74* is involved in osmatic stress and ABA signaling through interacting with CTD phosphatase-like 3 (CPL3) and CPL4 [57].

Taken together, all above analyses indicate that differential expression of genes in the brown module may cause differential responses to cold treatment between TT and YT.

### Roles of hub transcription factors (TFs) in cold stress

**Fig. 6:**
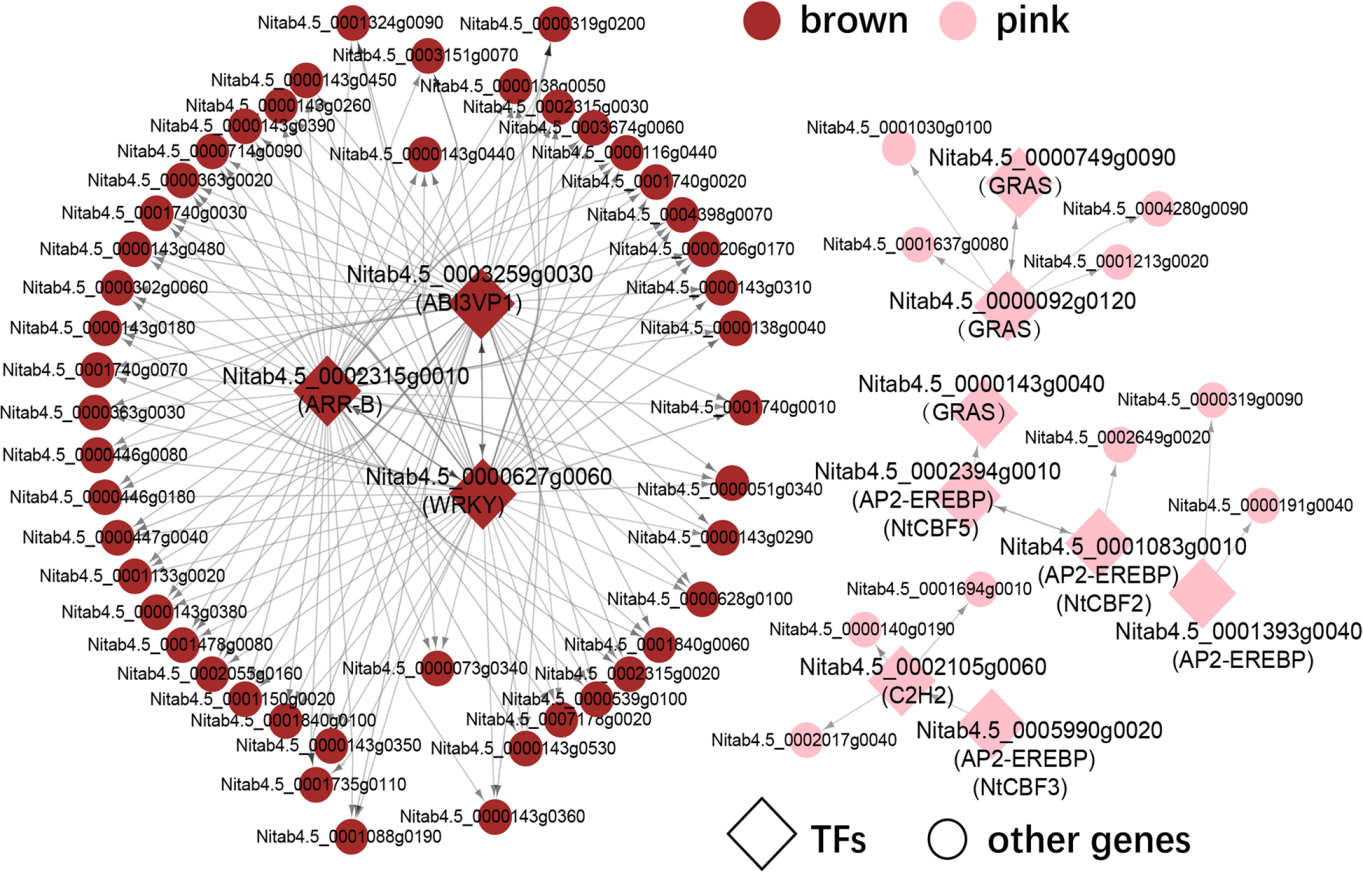
Genetic regulatory networks (GRNs) between hub genes. The circle represents gene, the diamond represents TF, and the connecting line is divided into source and arrow. The gene pointed by the arrow represents the gene targeted by source transcription factor. If both ends of the connecting line are arrows, the two TFs regulate each other

Based on WGCNA, we identified some important hub genes that were co-expressed in the same module with each other. We speculate if they have certain regulatory relationships with each other, and they play important roles in the regulation of DEGs under cold treatment. By using tree-based methods [58], we generated inferring regulatory networks to study the regulatory relationship between TFs and DEGs by using 11 hub TFs in the pink or brown module as regulators. We obtained genetic regulatory networks (GRNs) containing 11 TFs through the R package GENIE3 (Additional file 1: Fig. S12, Additional file 2: Table S7). We found that 11 TFs had possible interaction relationships with the aforementioned hub genes, but also with genes within other modules (Additional file 1: Fig. S12), indicating that 11 TFs play vital regulatory roles in responding to cold stress in TT or YT. To visualize the regulatory relationship among these genes more clearly, we exemplified the network only containing the hub genes/TFs of the pink or brown module identified in WGCNA (Fig. 6). We observed clearly regulatory relationships occurred between hub TFs and hub genes. For instance, in the brown module, the three hub TFs (ABI3/VP1, ARR-B and WRKY) exhibited regulatory relationships with hub genes or with each other (Fig. 6). It has been documented that WRKY TF family is involved in cold stress in several plants [59], such as *Arabidopsis* [60] and eggplant [61]. Moreover, regulatory relationships between TFs and hub genes also occurred in the pink module, including cold-responsive TFs *NtCBF2*, *NtCBF3* and *NtCBF5*, which were well characterized in tobacco [41]. Some of TFs (*GRAS*, *C2H2* and *AP2-EREBP*) associated with stress/stimulus responses were also detected in the network and exhibited regulatory relationships with other hub genes and DEGs (Fig. 6; Additional file 1: Fig. S12). In *Arabidopsis*, *GRAS* gene is stress-inducible and functions in enhanced cold tolerance via regulating the expression of other related genes [62]. Thus, according to GRN, we identified genes already known to be associated with cold stress in tobacco, such as *NtCBF* genes; more importantly, we also identified other genes that were involved in cold responses.

**Fig. 7:**
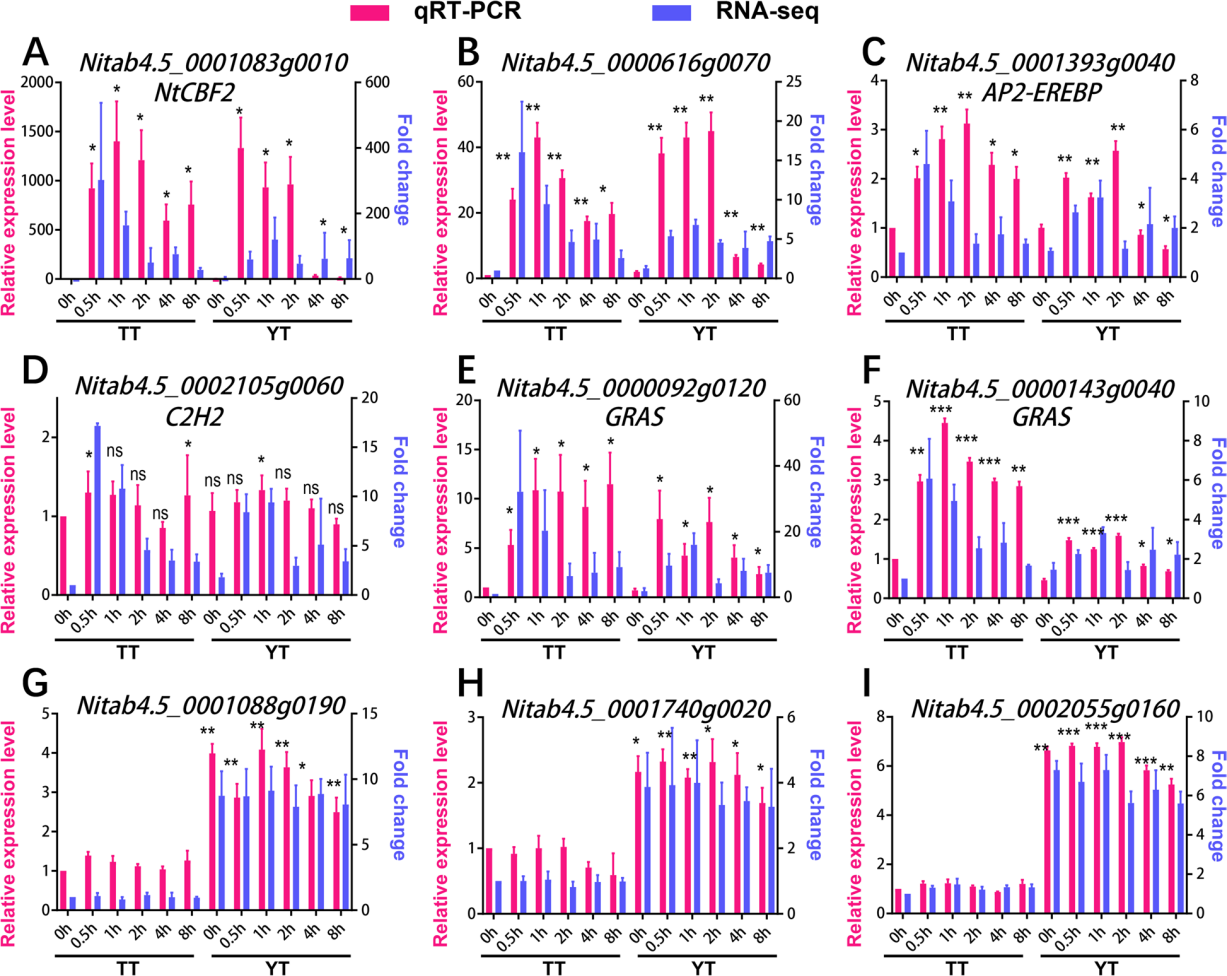
qRT-PCR assay of some key genes identified in the network. A dual y-axis plot illustrating in parallel comparisons of each individual gene examined detected by RNA-seq (expression fold change compared to 0 h of TT, blue) and qRT-PCR (relative expression levels, red). The right coordinate (y axis) with blue represents the expression fold change of RNA-seq, the left coordinate (y axis) with red represents the relative expression levels of qRT-PCR. The transcription levels of each gene in each time point were normalized relative to the internal control (*NtActin7*). Relative expression levels of genes examined were calculated and expressed as 2^-ΔΔCT^ [63] relative to the expression levels of the corresponding *NtActin7*, which were set as 1.0. The mean expression levels were calculated from three biological replicates. Error bars are standard deviations of three biological replicates. Significance test was determined using Student’s t test. “ns”: not significant, “*”: *p* < 0.05, “**”: *p* < 0.01, “***”: *p* < 0.001

To verify the accuracy of DEGs identified by RNA-seq data and hub genes identified through the GRNs, we conducted qRT-PCR assay for 13 randomly selected DEGs, including 6 hub genes /TFs in the pink module (Fig. 7A, B, C, D, E, F), and 7 hub genes/TFs in the brown module (Fig. 7G, H, I; Additional file 1: Fig. S14 A, B, C, D). We found that 9 genes (ca. 69%), including 6 (100%) genes in the pink module, 3 (ca. 43%) genes in the brown modules, exhibited a similar trend of expression change under cold treatment as compared to the results from RNA-seq (Fig. 7). For instance, *Nitab4.5_0001083g0010 (NtCBF2)* was up-regulated at all time points examined (0.5 h, 1 h, 2 h, 4 h, 8 h) as compared to the CK (0 h) (Fig. 7A), *Nitab4.5_0001088g0190* was up-regulated in YT relative to TT in each time point (Fig. 7G), while the remaining 4 genes were distinct from the RNA-seq results (Additional file 1: Fig. S14). Inconsistency between RNA-seq and qRT-PCR assay were most likely caused by the variations in different batch of biological samples with cold treatment. Even though we followed the same procedures as we did for RNA-seq for growing the material and cold treatment, it is hard to obtain 100% reproducibility at gene expression levels as before. We also cannot exclude the possibility that parts of DEGs identified by RNA-seq could be false positive, which are possibly not directly caused by cold treatment.

**Fig. 8:**
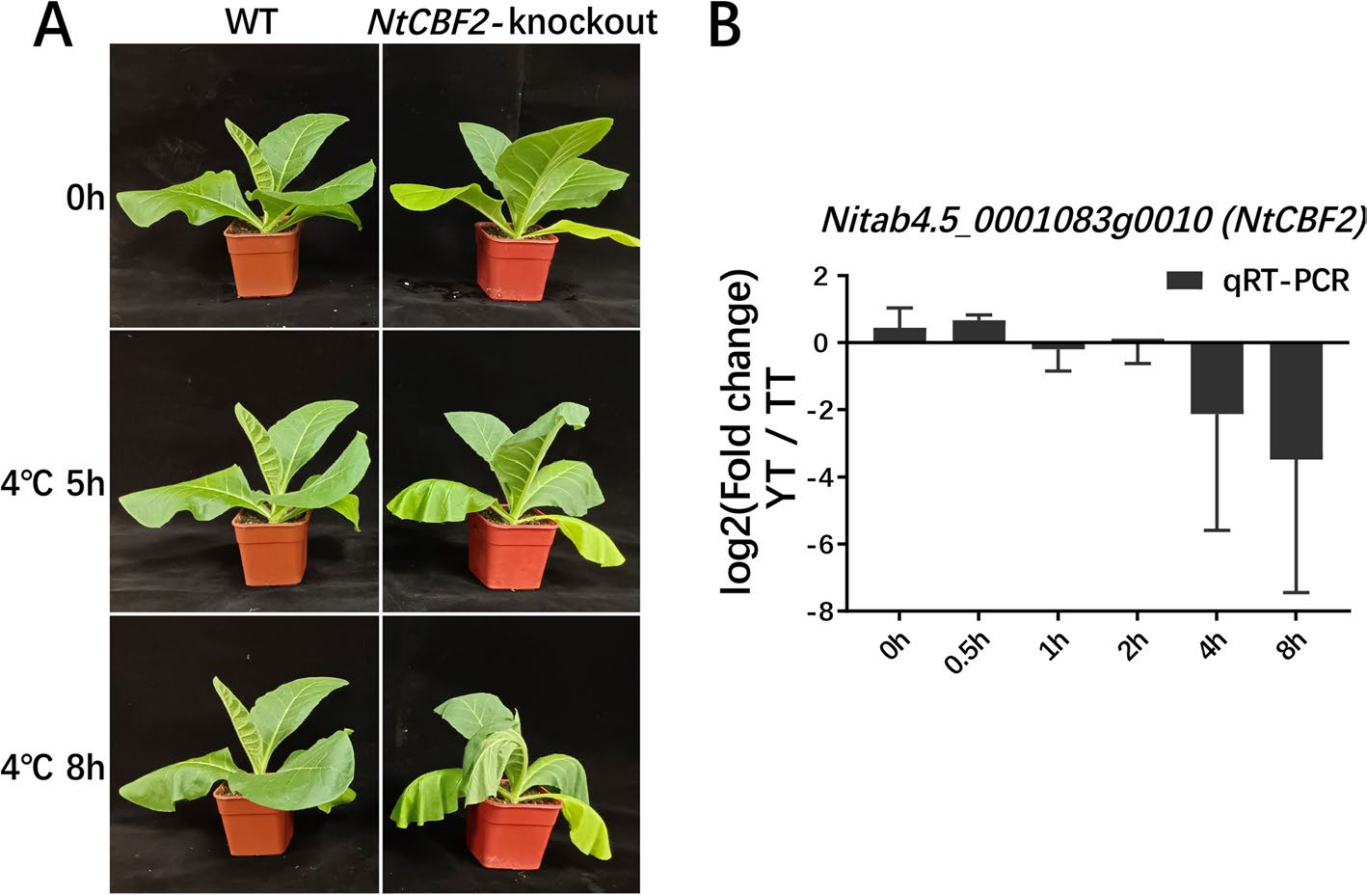
Experimental validation of *NtCBF2*. **A** CRISPR-Cas9 based validation of *NtCBF2* gene functioning in cold tolerance or responses. **B** The time course related differential expression of *NtCBF2* between TT and YT

To further confirm the role of *NtCBF2* in enhancing cold tolerance, we generated CRISPR-Cas9 based *NtCBF2* knockout (KO) transgenic line with K326 background (Fig. 8A, Additional file 1: Fig. S13). After conducting cold treatment, we found that the transgenic line with loss of *NtCBF2* gene function exhibited two droop leaves at 5 h post cold treatment, by contrast, the cold damage was even severe for extended cold treatment, since the transgenic line exhibited four droop leaves at 8 h post cold treatment as compared to the WT grown in the normal conditions. This result confirmed that *NtCBF2* gene can function in cold responses and tolerance, indicating the accuracy of constructed TF-centered regulatory network related to cold responses in tobacco. Furthermore, we compared the time course related differential expression pattern of qRT-PCR results for *NtCBF2* between TT and YT. We found that fold changes of its expression levels between YT and TT were increased at 0.5 h, 4 h and 8 h post cold treatment as compared to CK (0 h), while the cold-induced expression trend of *NtCBF2* was opposite between YT and TT at 0.5 h relative to 4 h/8 h (Fig. 8B). After cold treatment, *NtCBF2* was more expressed at 0.5 h, but much less expressed at 4 h and 8 h in YT than in TT (Fig. 8B). To assess if there exist any sequence variations resulting in differential expression of *NtCBF2* under cold treatment between two varieties, we conducted RT-PCR to obtain full CDS sequences, and conducted PCR to amplify the promoter region (1.5 kb upstream of ATG) from each variety for sequencing. We did not observe any sequence polymorphisms in the cDNA and promoter regions of *NtCBF2* between YT and TT (Additional file 1: Fig. S15). The underlying regulatory mechanisms need to be further investigated. The possible reasons related to cold inducible expression change of this gene between YT and TT were discussed in the discussion section.

## Discussion

To cope with a variety of environmental stresses adversely affecting plant normal growth and development, sessile plants have to adjust changes at multiple levels to adapt to these stresses for better survival. They include morphological, physiological, biochemical, metabolic, genetic and epigenetic changes [64–67]. Global transcriptional changes in response to cold stress have been intensively investigated in various plant species [22, 23, 32, 68–70]. However, transcriptomic network studies related to cold treatment are quite limited in tobacco. In this study, we conducted comprehensive multiple time-point transcriptomic analyses between cold-susceptible tobacco cultivar TT and cold-tolerance tobacco cultivar YT. We observed genes up- or down-regulated at each time point in each cultivar or between two cultivars (Additional file 2: Table S2), including cold-responsive genes or TFs such as protein kinases and *NtCBF* genes (Additional file 2: Table S6). Similar comparative transcriptomic studies related to cold treatment have also been conducted in two tobacco varieties with contrasting cold sensitivity [71], due to tobacco genome sequences unavailable at that time, 3557 cold induced DEGs were identified using the assembled all unique genes and 1840 DEGs were functionally annotated when using Potato (*Solanum tuberosum*) genome as reference. For a parallel comparison between two studies, we reanalyzed 1840 DEGs as we did by using tobacco (*Nicotiana Tabacum*) genome sequence as reference for gene annotations. We found that only 940 DEGs best matched in *Nicotiana Tabacum* genome. According to the overlapping comparison, we found that 354 out of 940 (ca. 38%) DEGs were also present in 9583 DEGs and 3 of them, *Nitab. 4.5_0000532G0070*, *Nitab. 4.5_0001244G0020* and *Nitab. 4.5_0002394G0010* (*NtCBF5*), were present in 100 hub genes in our study. Inconsistency between two cold related studies in tobacco is most likely caused by using different reference genomes for identification of DEGs and subsequent gene annotation.

Moreover, our study also provided evidence showing transcriptional changes between TT and YT, which possibly explains transcriptome-based differential responses to cold stress between TT and YT. It provided evidence showing common mechanisms involved in cold responses between TT and YT. *NtCBF* genes, cold-responsive genes, were up-regulated in both TT and YT. In particular, TT and YT share similar co-expression gene networks (the pink module, Fig. 5B). We also observed genotype-related gene expression changes under normal growth condition (CK). There were 857 up-regulated genes and 750 down-regulated genes in YT compared to TT before cold treatment (Fig. 2A). Moreover, YT displayed less gene expression changes than TT under cold treatment (Additional file 2: Table S2), thus YT can spend less energy to remodel chromatin structure for reprogramming gene transcription than TT. We assume that those DEGs before cold induction may help to enhance YT to tolerate cold stress at initial stages in response to cold stress. In addition, we found that TT-specific up-regulated genes including gene upregulated in TT but down-regulated in YT exhibited more GO term enrichment associated with various biological and metabolic processes and stress responses. Plants need to consume more energy to complete those processes, thus have to use less energy to cope with cold stress, therefore resulting in sensitivity to cold treatment. Eukaryotic cells need to spend energy from oxidation of metabolic fuels to accomplish all kinds of energy-dependent biological processes [72]. Peroxisome with ATP supply is essential for seedling development in *Arabidopsis* [73]. By contrast, YT-specific up-regulated genes including gene up-regulated in YT but down-regulated in TT mainly function in photosynthesis, membrane functions, and production of energy. Thus, YT can produce more energy and maintain membrane stability to facilitate the plants to endure cold injury. It has been reported that plants with cold acclimation have increased photosynthetic capacity [74–78]. Remodeling of membrane proteins and lipids and maintenance of membrane fluidity and permeability are one of the principal adaptation strategies for plants accommodating to cold stress [79–81].

In addition, we observed differential co-expression network between TT and YT. Genes in the brown module with GO terms enriched in stress responses were up-regulated in YT instead of TT (Fig. 5C, Additional file 1: Fig. S11B). More importantly, we identified 3 TF (ABI3/VP1, ARR-B and WRKY) mainly functioning in differential cold responses between two cultivars, and 3 key TFs (GRAS, AP2-EREBP and C2H2) primarily involved in cold responses (Fig. 6). Some of them have been well documented to be involved in cold or stress responses in other plant species. For example, C2H2 zinc finger gene family (ZAT12 and ZAT10) have been reported to be involved in stress responses [82, 83]. Tobacco GRAS are homologous to SCL13 and PAT1 in *Arabidopsis*, which are involved in phytochrome A (PhyA) signal transduction and B (PhyB)-related environmental temperature responses, respectively [84–86]. Ethylene responsive element binding factors (ERFs) are involved in various abiotic stresses, such as cold, drought and salinity [87]. COBL7 gene has been reported to function in cellulose biosynthesis in *Arabidopsis* and rice [88, 89]. The involvement of AP2-EREBP families in stress responses have been documented in a couple of plants, such as *Arabidopsis* [90], rice [91], maize [92], cotton [93], *Brachypodium Distachyon* [94], *Ammopiptanthus nanus* [95]. CERK1 has been reported to mediate roles of chitin in salt stress in *Arabidopsis* [96]. AtWRKY21 and ARR 12 have been reported to act as negative regulators during drought stress in *Arabidopsis* [52, 97]. Salinity stress can increase expression of PSP in *Brassica juncea* [51]. Ser/Thr phosphatase type 2A (PP2A) plays crucial roles in the regulation of adaptive biotic and abiotic stress responses in plants [98]. *Arabidopsis* transgenic plants with overexpression of AB13/VP1 gens (*MtRAV*3) from *M. truncatula* increase tolerance to cold treatment [99]. Rice transgenic plants with overexpression of *GSA1* encoding a UDP-glucosyltransferase can enhance tolerance to heat, NaCl and PEG stresses [56]. Our study also showed that proteinase kinases were differentially expressed under cold stress, suggesting that protein kinases can potentially act as key regulators in response to cold treatment in tobacco. Protein kinases have been reported to play vital roles in cascading signal transduction during abiotic stresses in plants [100, 101]. Maize plants with constitutive expression of tobacco mitogen-activated protein kinase kinase kinase (*Nicotiana PK1*) exhibited enhanced freezing resistance [102]. Tobacco plants with overexpression of *Malus domestica* calcium-dependent protein kinases (*MdCPK1a*) gene had low levels of ROS accumulation, resulting in increased cold resistance [103]. The roles of protein kinases in cold responses in tobacco need to be further investigated.

In addition, it is worth noting that *NtCBF2* was selected through WGCNA using DEGs between TT and YT, but was only functionally validated in K326 background instead of either cultivar examined. Higher expression of *NtCBF2* at the early stage of cold treatment may facilitate YT to enhance cold resistance than TT. This was evidenced by the qRT-PCR results that *NtCBF2* was more expressed at 0 h (before cold treatment) and 0.5 h post cold treatment but was much less expressed at 4 h and 8 h after cold treatment in YT as compared to TT (Fig. 8B). Thus, YT may activate cold responses earlier, thereby being subjected to less cold damage than TT at the beginning of cold treatment. In addition to *NtCBF2*, YT had more cold responsive genes with higher expression than TT after cold treatment (Fig. 5). Thus, differential cold responses between two varieties are possibly caused by differential expression of some of key cold responsive genes or potential difference in functions of their downstream targets of cold responsive TFs.

Given that no sequence polymorphisms occurred in the cDNA and proximal promoter regions of *NtCBF2* between YT and TT (Additional file 1: Fig. S15), differential transcription of *NtCBF2* between YT and TT could be caused by several possibilities: 1) differential expression of its upstream regulators such as TFs; 2) sequence variations occurred in the distal cis-regulatory elements (CREs) affecting the binding of the regulatory protein or protein complex; 3) occurrence of distinct epigenetic mechanisms, such as DNA methylation, histone modifications and chromatin openness. Further evidence needs to be provided. Epigenetic characterization like DNA methylation and histone modifications may help to reveal possible mechanisms responsible for differential expression of cold responsive genes like *NtCBF2* under cold treatment between YT and TT.

## Conclusions

Collectively, through comprehensive multiple time-point transcriptomic and network analyses, we identified key genes and transcription factors that contribute to differential cold tolerance of two tobacco cultivars. Thus, our study provides valuable transcriptomic resources for studying cold adaptation mechanisms in plants. It helps to identify key cold responsive genes for genetic modifications of tobacco cultivars for improving cold/freezing tolerance.

## Materials and methods

### Plant growth and cold treatment

Two cultivated tobacco (*Nicotiana tabacum* L.) varieties with contrasting cold responses, Taiyan8 tobacco (TT, cold susceptibility) and Yanyan97 tobacco (YT, cold resistance) provided by Drs. Risheng Hu and Yangyang Li from Hunan Tobacco Research Institute, were used in this study. The seeds were sterilized in 75% ethanol for 10 min at room temperature (RT) and washed with sterile water for five times. Sterilized seeds were germinated and grown in 1/2 MS medium in an illuminated incubator (16/8 h day/night light cycle, 26/24 °C day/night temperature setting with 70% relative humidity). Tobacco seedlings at the five-leaf stage were used for cold treatment at 4 °C. Leaves were collected from CK and cold-treated plants at different time points (0, 0.5 h, 1.0 h, 2.0 h, 4.0 h and 8.0 h). Collected samples were immediately frozen in liquid nitrogen for RNA preparation. Each sample was biologically triplicated for transcriptomic analyses.

### RNA-seq library preparation and data analyses

Total RNA was prepared from tobacco leaves using TRIzol Reagent (ThermoFisher Scientific, Cat # 15596026). After complete removal of genomic DNA contamination using DNase treatment, total 10 μg purified RNA was used for mRNA enrichment using poly(T)-conjugated magnetic beads. mRNA-seq libraries were constructed using an Illumina TruSeq RNA Sample Prep Kit following the manufacturer’s manual. All libraries were sequenced on Illumina Hiseq4500 platform.

The raw sequence data were processed to remove low-quality reads using FastQC (v0.11.5) and Trim_Galore (v 0.6.4). The filtered high-quality reads were mapped to the Nicotiana tabacum genome [104] (ftp://ftp.solgenomics.net/genomes/Nicotiana_tabacum/edwards_et_al_2017/annotation/) using HISAT2 (v2.0.5) with default parameters. Reads with a mapping quality below 20 were excluded for further analyses using SAMtools (v1.9). StringTie (v1.3.4d) and FeatureCounts (v1.6.4) were used to obtain transcripts per million (TPM) and read counts for all annotated genes in the tobacco genome. Correlation between the biological triplicates was determined using Pearson correlation coefficient with TPM value.

A Principal Component Analysis (PCA) was performed using prcomp utilities in the R package. Differential gene expression levels were analyzed using DESeq2. Genes with expression levels of |log_2_^(fold change)^| > 1 and padj. < 0.05 were retained for downstream analyses. Gene Ontology (GO) enrichment analyses were conducted by using the online website AgriGO (http://systemsbiology.cau.edu.cn/agriGOv2/specises_analysis.php?&SpeciseID=15& latin=Nicotiana_tabacum), which is specifically designed for agricultural species.

### qRT-PCR assay

For qRT-PCR assay, total RNA was extracted from the samples with similar treatment as we did for RNA-seq. The extracted RNA was treated with DNase for removal of genomic DNA contamination followed by reverse-transcription for synthesis of the first cDNA strand. qRT-PCR was conducted using the SYBR mix. *NtActin7* was used as an internal control. Relative expression levels of genes examined were calculated from three independent biological replicates and expressed as 2^-ΔΔCT^ [63]. Primers for qRT-PCR assay are listed in Additional file 2: Table S9.

### Identification of *NtCBF* genes

For identification of cold-responsive *NtCBF* genes, We followed the *NtCBF* gene named by dehydration-responsive element-binding (DREB) protein in the previous literatures [41]. To extract the corresponding gene id, the DREB protein sequences were further aligned to *Nicotiana tabacum* protein genome (ftp://ftp.solgenomics.net/genomes/Nicotiana_tabacum/edwards_et_al_2017/annotation/) using Protein-Protein BLAST (v2.9.0+). Detailed information of 21 CBF genes is listed in Additional file 2: Table S6.

### Generation of the K326 transgenic line

The *Nitab4.5_0001083g0010.1*(*NtCBF2*) gene identified in the GRN was chose for preparing a CRISPR-Cas9 vector (Additional file 2: Table S9). The vector was used for generation of transgenic plant using leaf disc transformation as the published procedures [105]. Homozygous transgenic plants were screening through sequencing and grown in the growth chamber for collecting more seeds for the cold treatment experiments.

### Co-expression network analyses

Weighted Gene Co-Expression Network Analysis (WGCNA) package [50] was used in R for co-expression network analyses. The 9583 DEGs with TPM values greater than 1 at least at one time point in two cultivars were used for WGCNA (https://horvath.genetics.ucla.edu/html/CoexpressionNetwork/Rpackages/WGCNA/Tutorials/). A convenient 1-step network construction and module detection function were used for generation of co-expression gene network and identification of each module with distinct functions. The eigenvalues were used to indicate association of genes between each module and at each time point within each cultivar. All co-expressed DEGs were grouped into 17 cultivar- and time-point-specific modules. Each network was visualized using Cytoscape (v.3.7.2).

### Genetic regulatory networks (GRNs) analyses

We followed the user manual of the R package GENIE3 (v1.12.0), (https://bioconductor.riken.jp/packages/release/bioc/vignettes/GENIE3/inst/doc/GENIE3.html) [58], to generate a regulatory network containing 9583 DEGs at each time point in the two varieties and 11 identified hub TFs as regulators. Subsequently, we set the threshold (weight > =0.2) to obtain a regulatory network diagram with a strong regulatory relationship between 11 TFs and 3308 DEGs (Additional file 1: Fig. S12) (See Additional file 2: Table S7 for detailed information of the regulation network diagram). The GRNs were visualized using Cytoscape (v.3.7.2).

### Statement of plant material used in the study

Study protocol must comply with relevant institutional, national, and international guidelines and legislation.

## Supplementary Information

**Additional file 1.**

**Additional file 2.**

## Data Availability

The raw RNA-seq data used in this study were deposited in the NCBI Gene Expression Omnibus (GEO) (http://www.ncbi.nlm.nih.gov/geo/) with accession number GSE173352. The materials of this study were provided by Hunan Tobacco Research Institute. A voucher specimen of this material has not been deposited in a publicly available herbarium. Correspondence and requests for materials should be addressed to Wenli Zhang (wzhang25@njau.edu.cn) or Risheng Hu (495298768@qq.com).

## Abbreviations

ABI3/VP1: ABSCISIC ACID INSENSITIVE 3/VIVIPAROUS 1, RAV
ARR: Type A response regulator
CBF: C-REPEAT BINDING FACTOR
COR: Cold Regulated
CPL: CTD phosphatase-like
DEGs: Differentially expressed genes
DREB: Dehydration-responsive element-binding
ERFs: Ethylene responsive element binding factors
GO: Gene Ontology
GRNs: Genetic regulatory networks
PCA: Principal Component Analysis
PKs: Protein kinases
PP2A: Ser/Thr phosphatase type 2A
PSP: 3-phosphoserine phosphatase
RT: Room temperature
TPM: Transcripts per million
TFs: Transcription factors
TT: Tai tobacco
WGCNA: Weighted Gene Co-Expression Network Analysis
YT: Yan tobacco
