# Supplementary material for: Transcriptome-based gene regulatory network analyses of differential cold tolerance of two tobacco cultivars
Source: BMC Plant Biol. 2022 Jul 26;22:369. doi: 10.1186/s12870-022-03767-7 (PMC9316383; doi:10.1186/s12870-022-03767-7)
Supplement: Supplementary file 1 — Additional file 1. [file 12870_2022_3767_MOESM1_ESM.pdf]

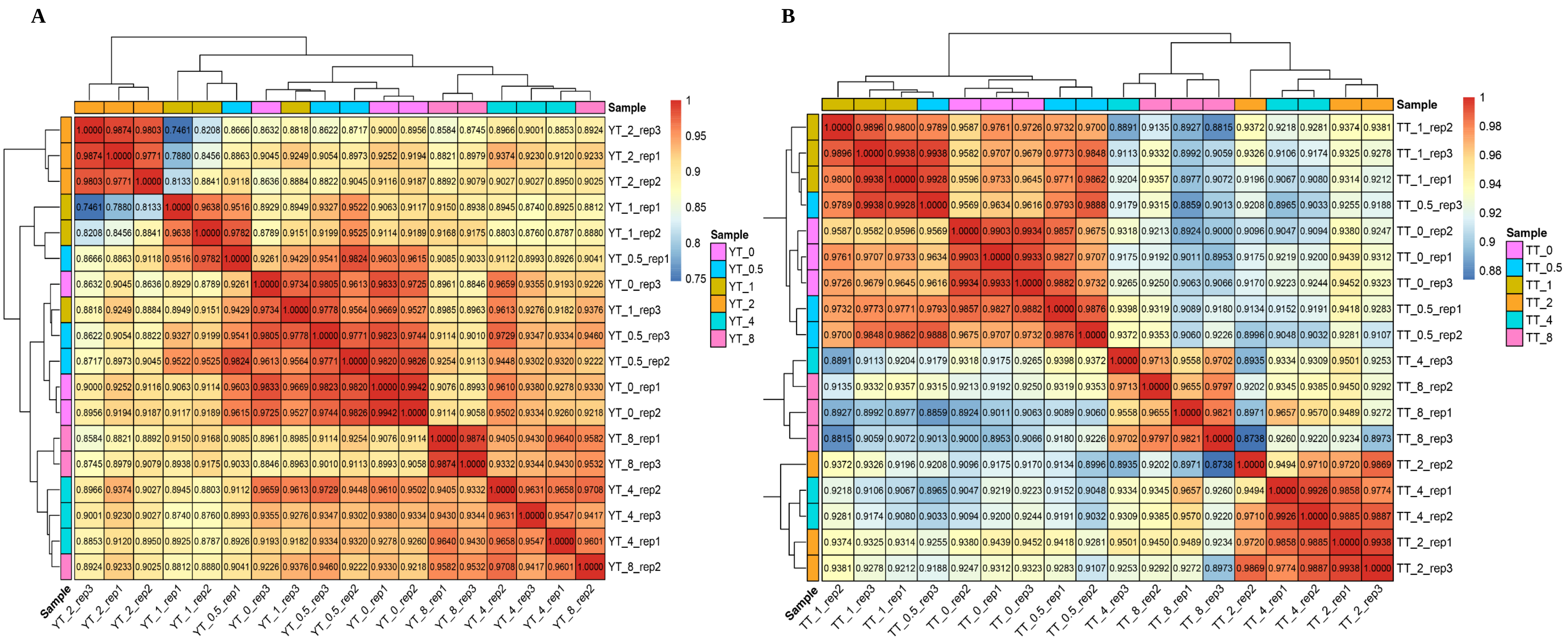

Additional file 1: Fig. S1 Hierarchically clustered correlation matrix of RNA-seq data generated from YT (A) and TT (B) subjected to cold treatment at different time points. Three biological replicates for each time point. Pearson correlations were calculated and hierarchically clustered by the heatmap package in R.

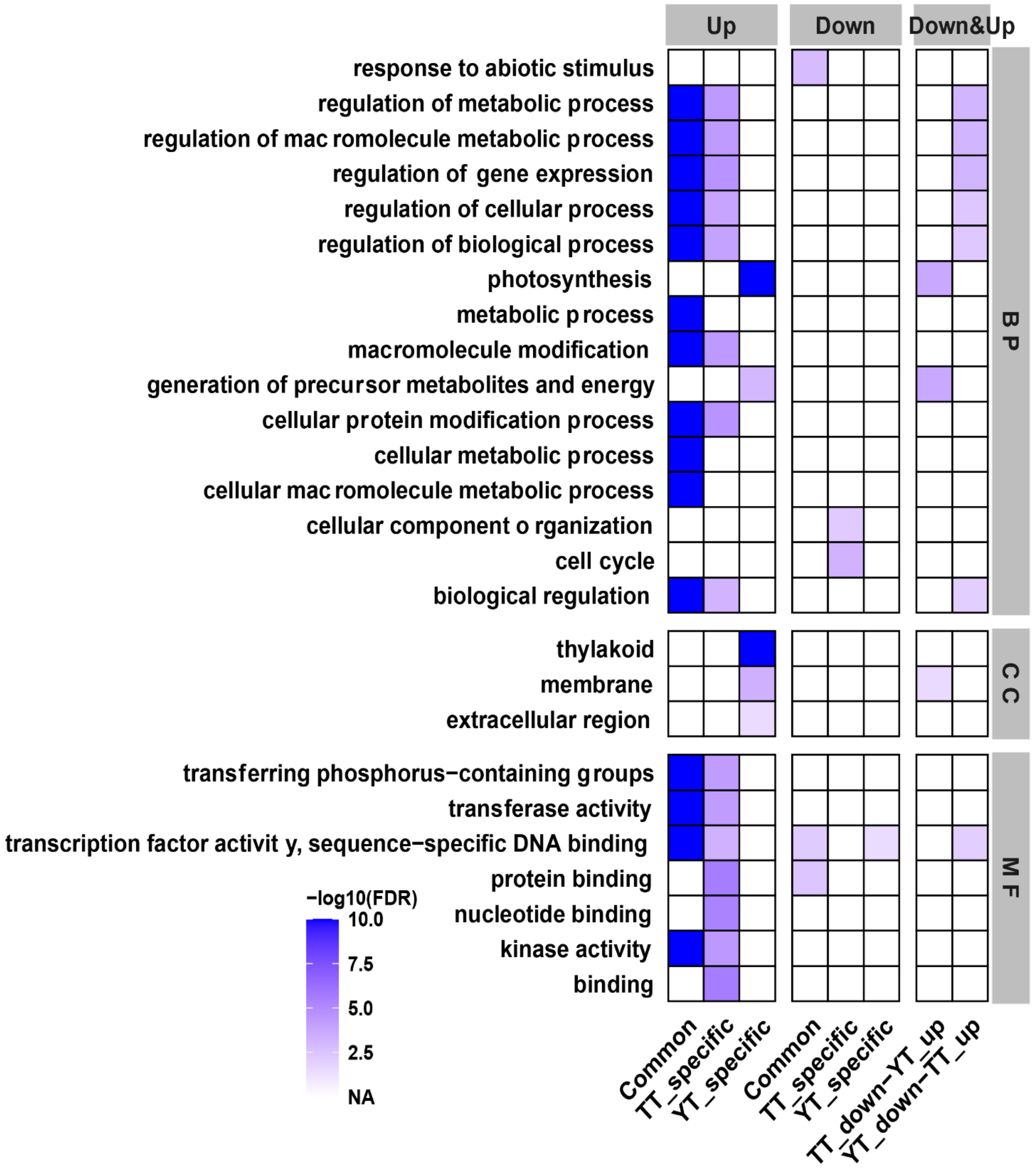

Additional file 1: Fig. S2 GO term enrichment analyses of up-regulated or down-regulated genes that were specific for YT or TT or shared between YT and TT.

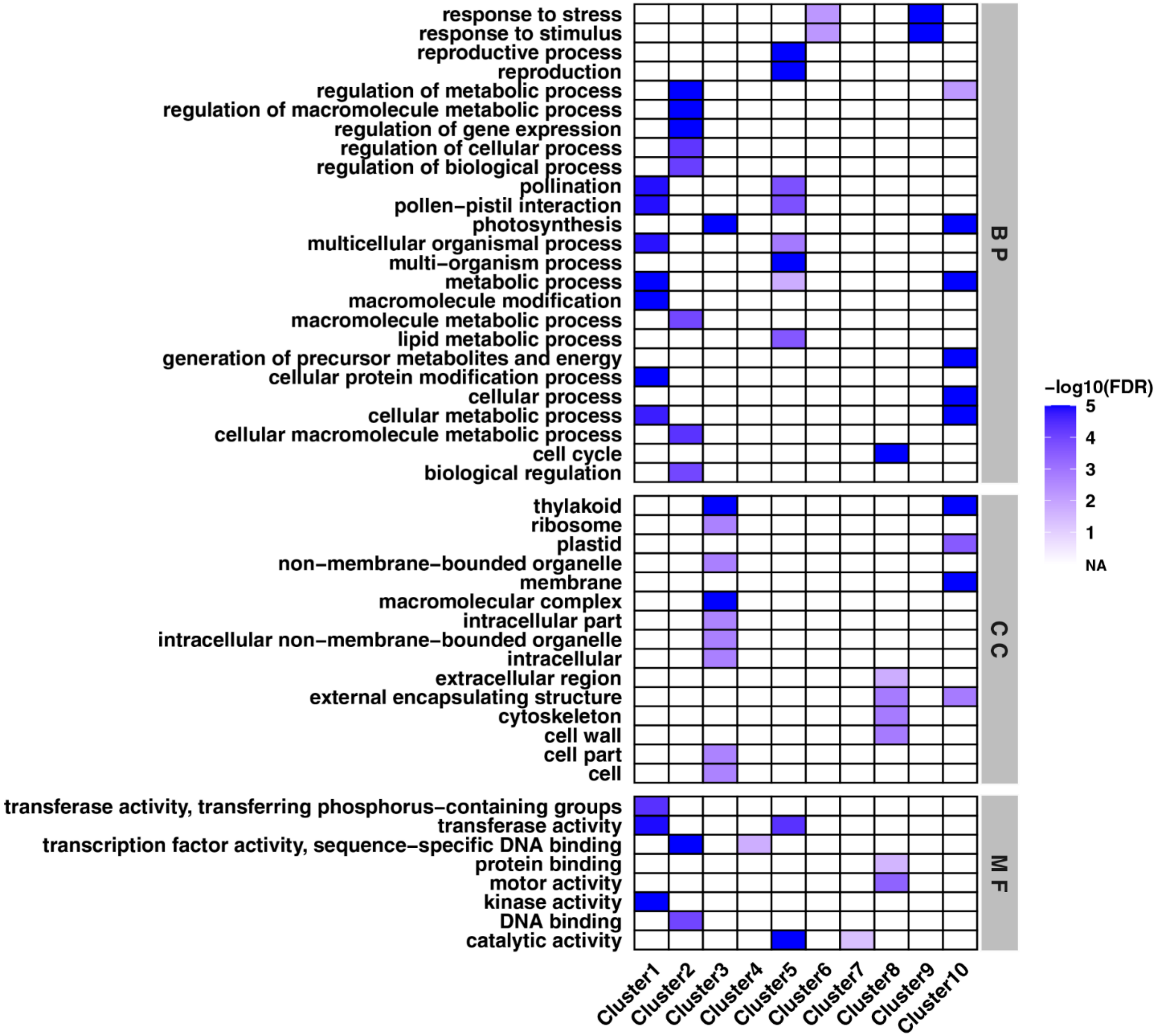

Additional file 1: Fig. S3 GO term enrichment analyses of each cluster in Fig.2B.

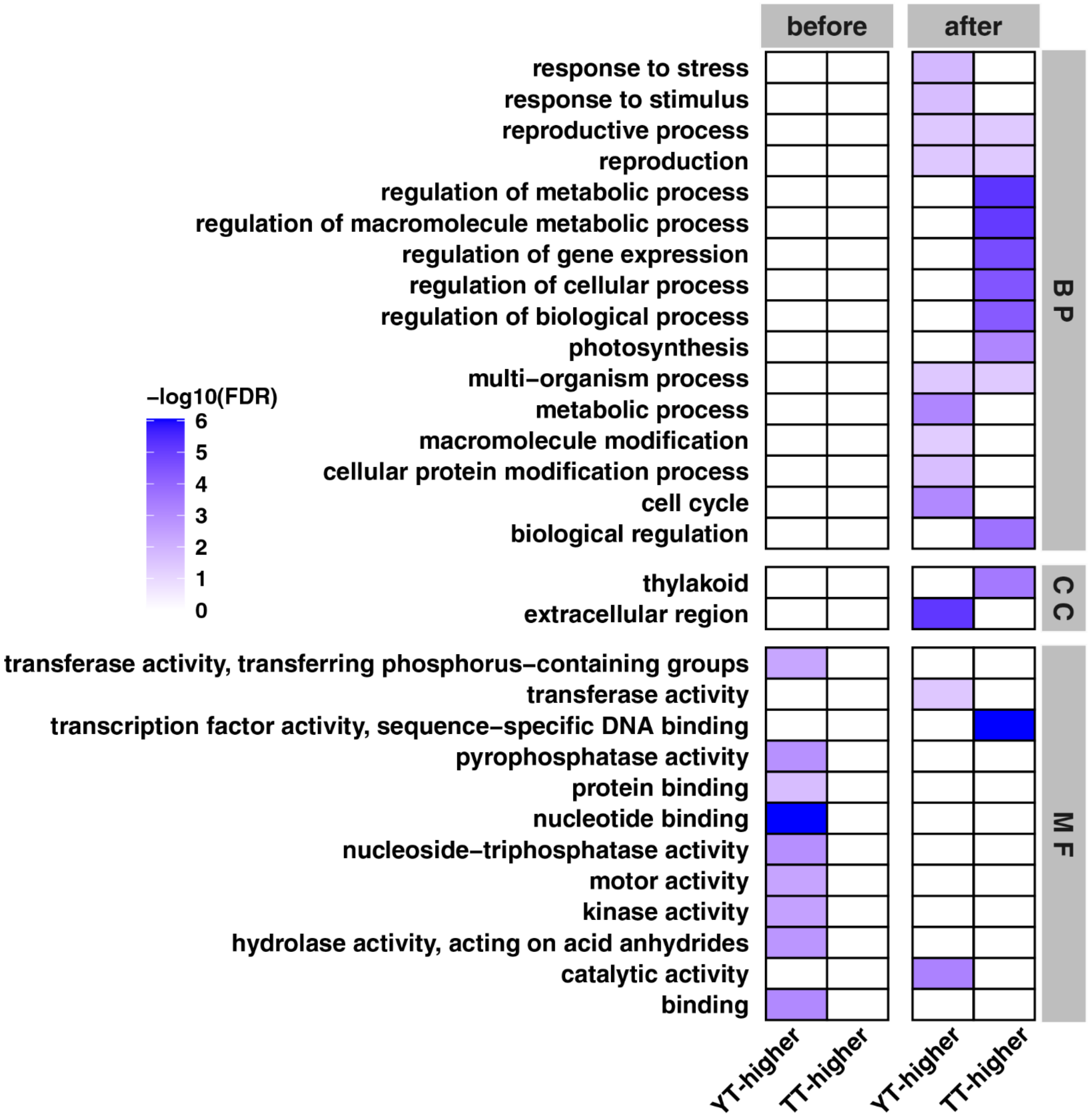

Additional file 1: Fig. S4 GO term enrichment analyses of genes that were up-regulated or down-regulated before (CK) or after cold treatment.

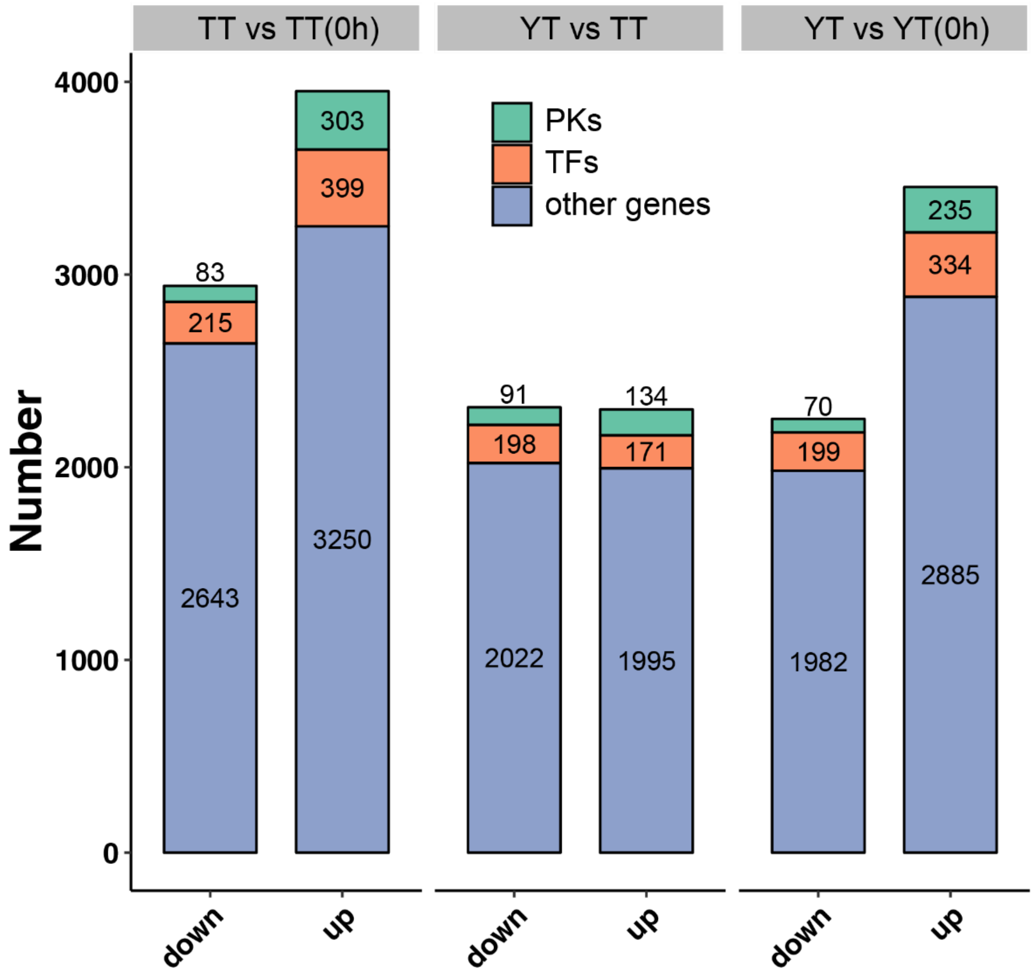

Additional file 1: Fig. S5 Bar plots showing the number of genes, TFs and PKs differentially expressed at least at one time point in TT, YY or between YT and TT before (0 h) and after cold treatment (0.5 h, 1 h, 2 h, 4 h, 8 h).

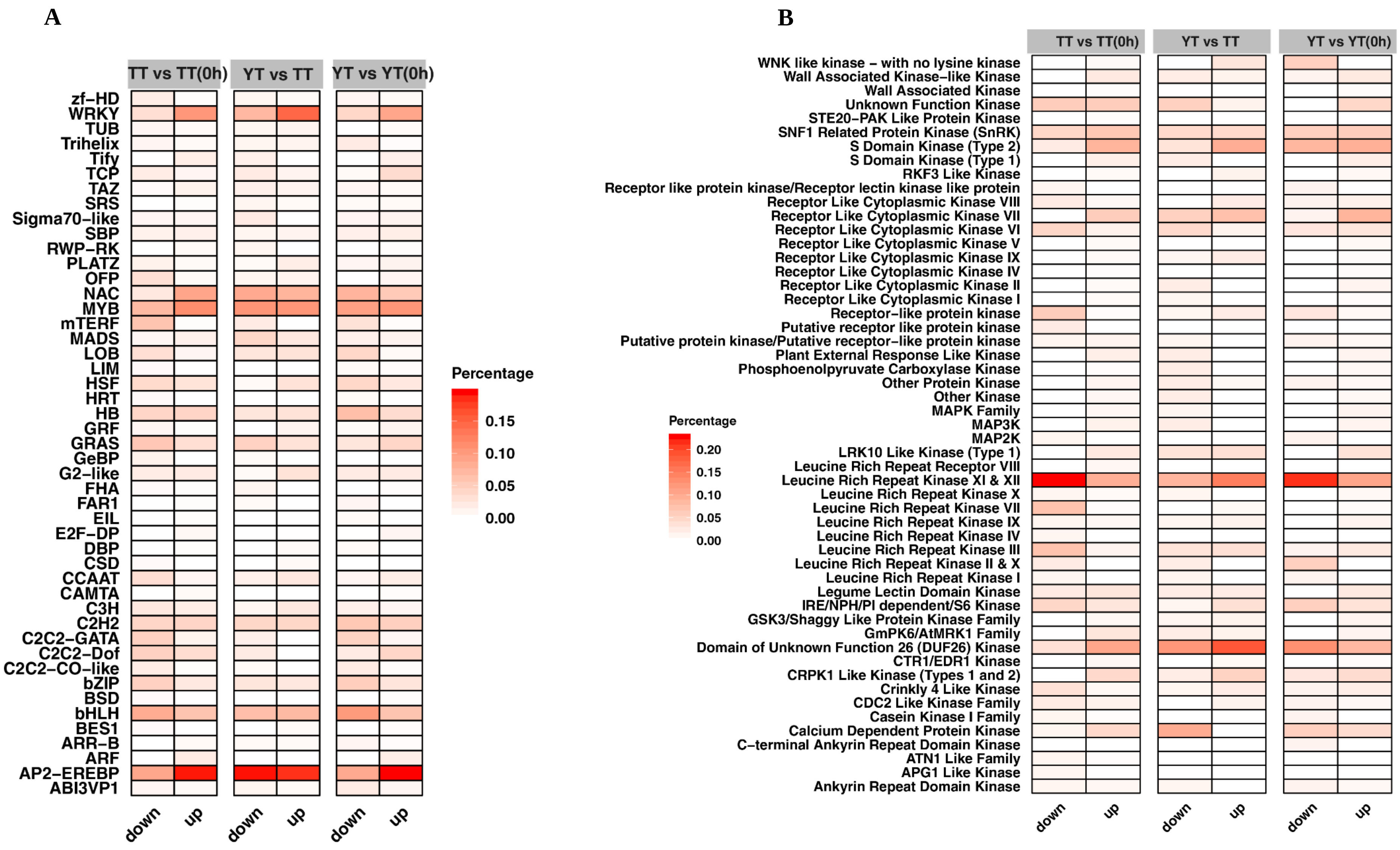

Additional file 1: Fig. S6 (A, B) Heatmap showing the type percentage of the TFs **(A)** or PKs **(B)** differentially expressed at least at one time point in TT, YY or between YT and TT before (0 h) and after cold treatment (0.5 h, 1 h, 2 h, 4 h, 8 h).

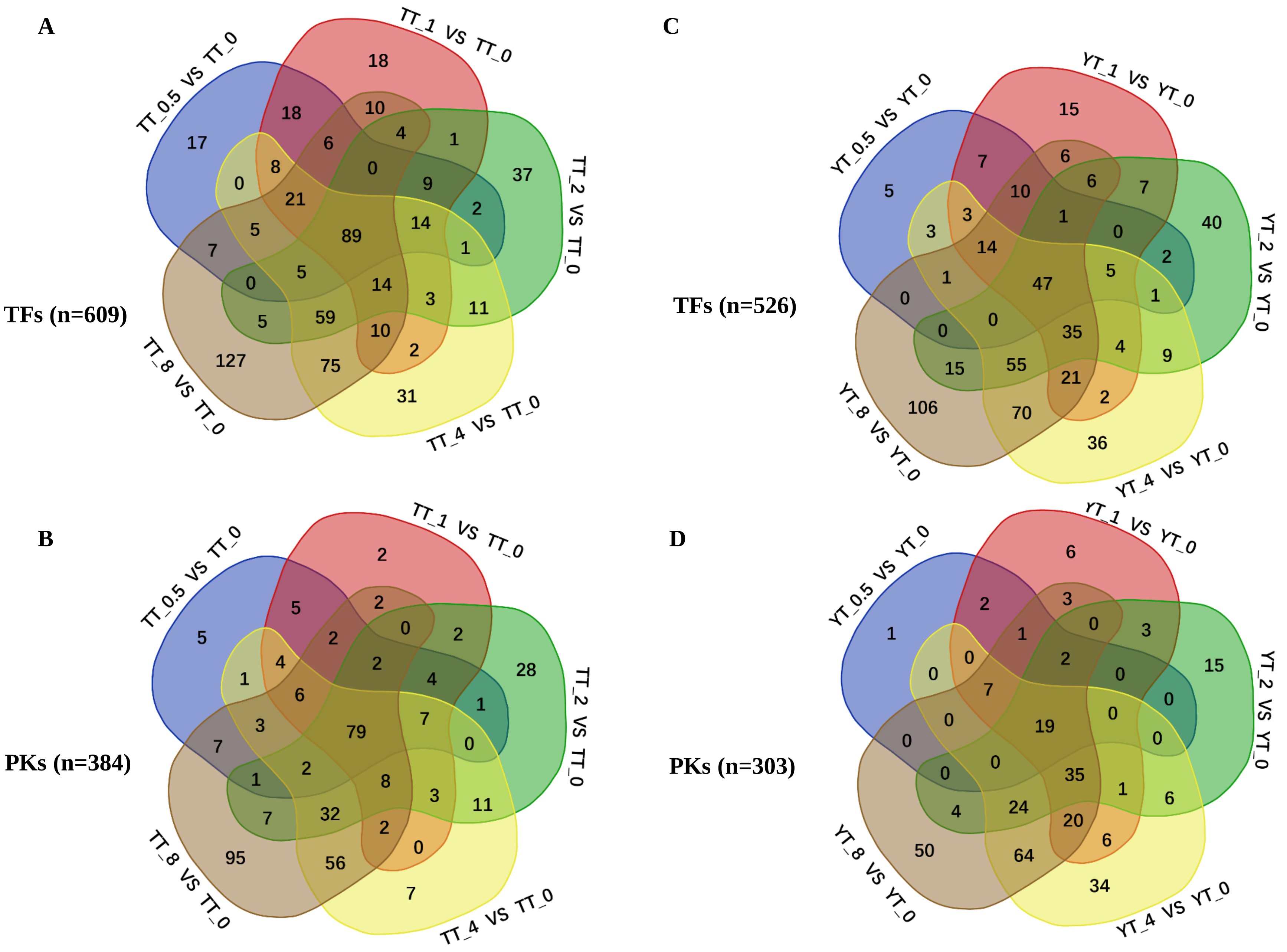

Additional file 1: Fig. S7 (A, B) Venn plots showing TFs **(A)** or PKs **(B)** differentially expressed at cold treatment (0.5 h, 1 h, 2 h, 4 h, 8 h) relative to 0 h in TT. **(C, D)** Venn plots showing TFs **(C)** or PKs **(D)** differentially expressed at cold treatment (0.5 h, 1 h, 2 h, 4 h, 8 h) relative to 0 h in YT.

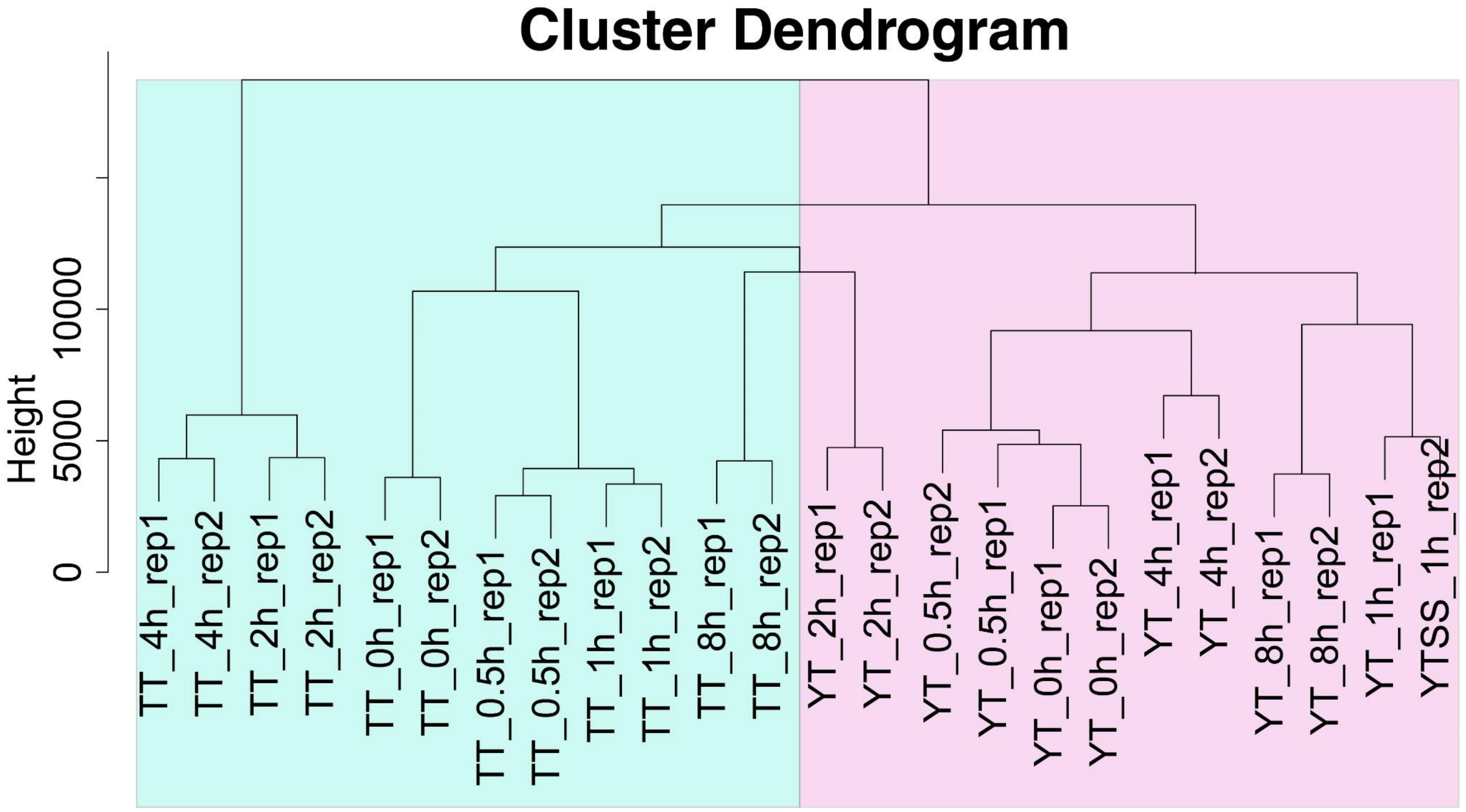

Additional file 1: Fig. S8 The clustering tree diagram shows different clustering groups.

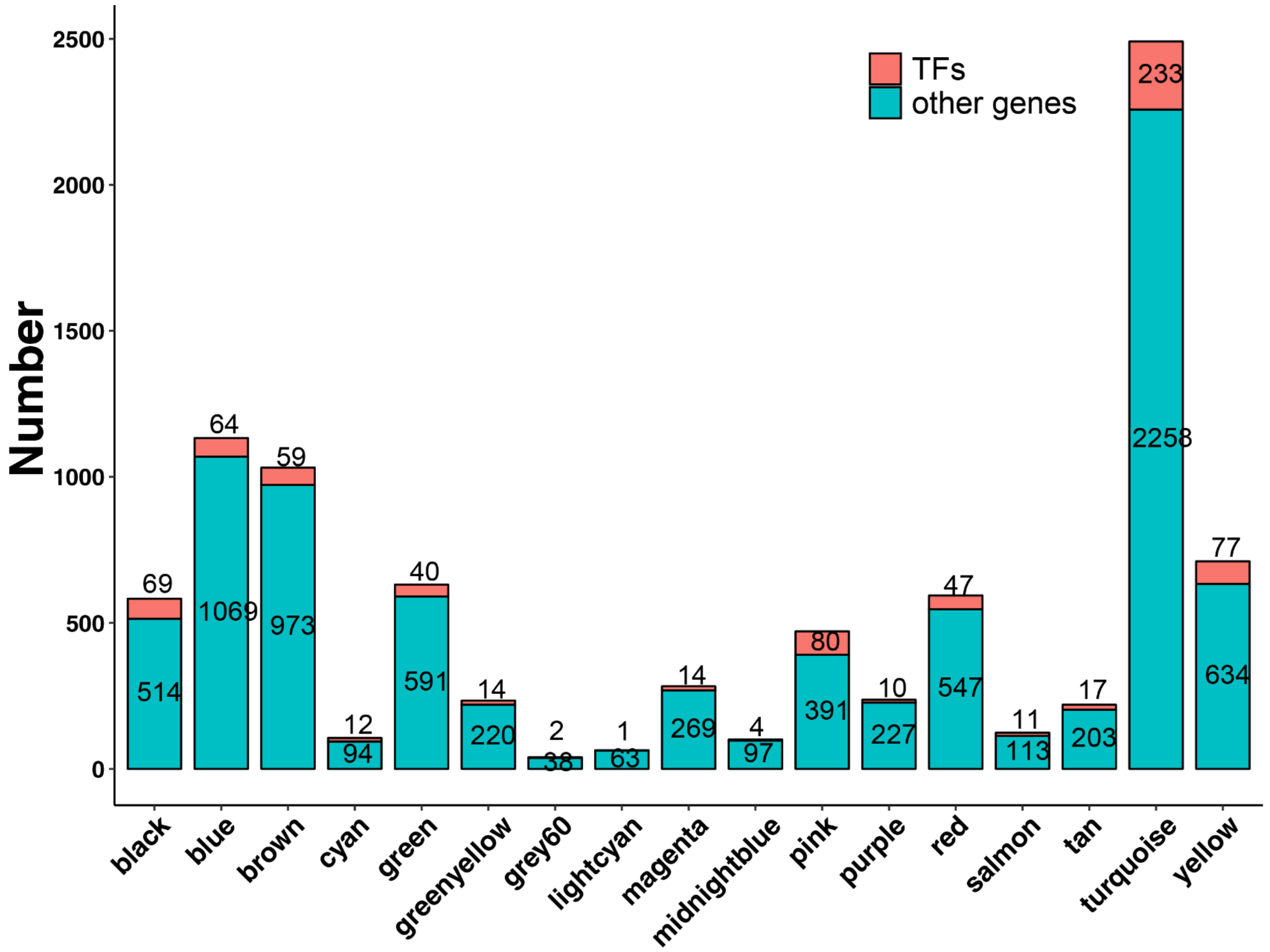

Additional file 1: Fig. S9 Bar plots showing the number of genes and TFs distributed in the 17 co-expression modules.

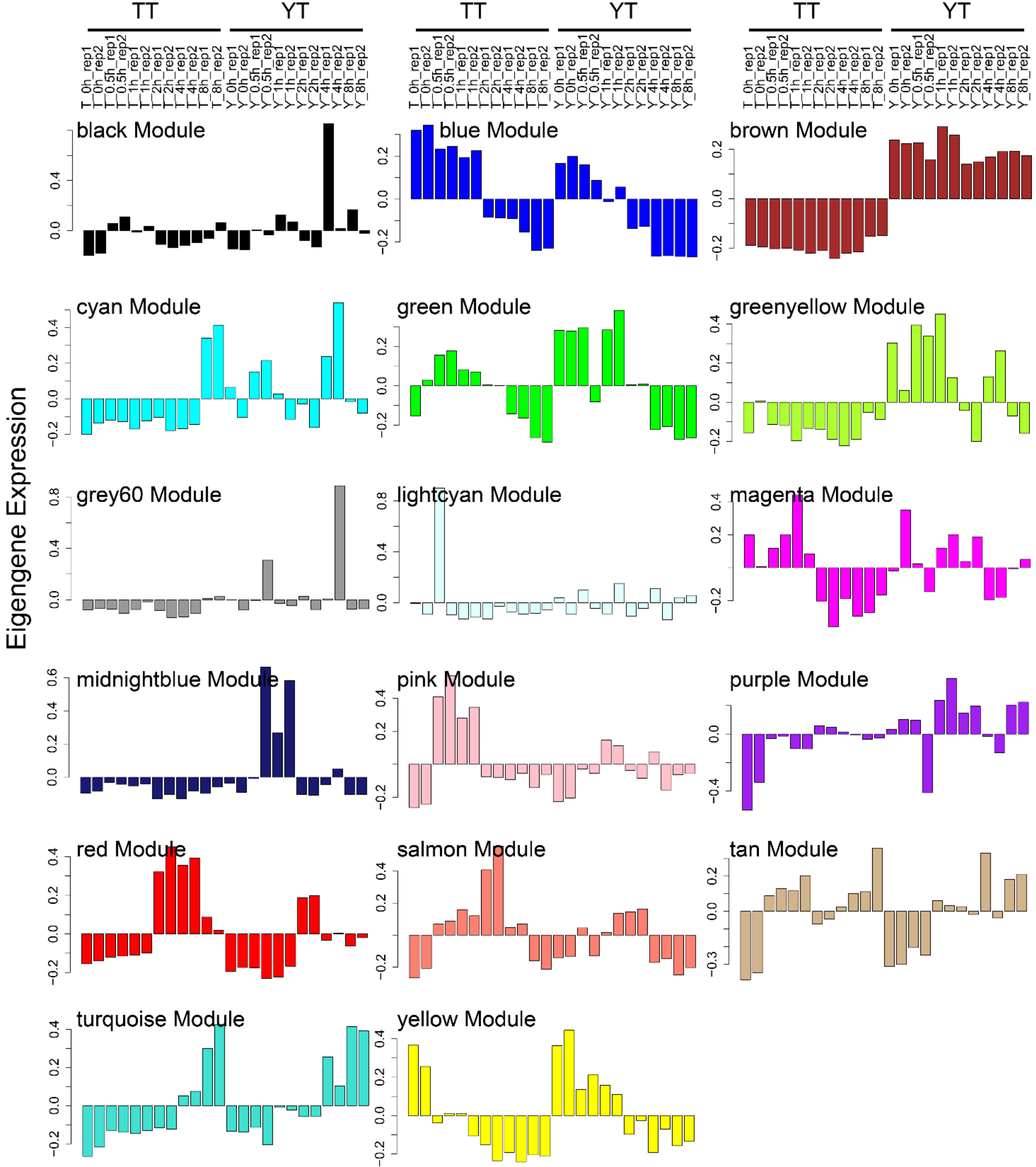

Additional file 1: Fig. S10 Module standardized gene expression pattern of each module.

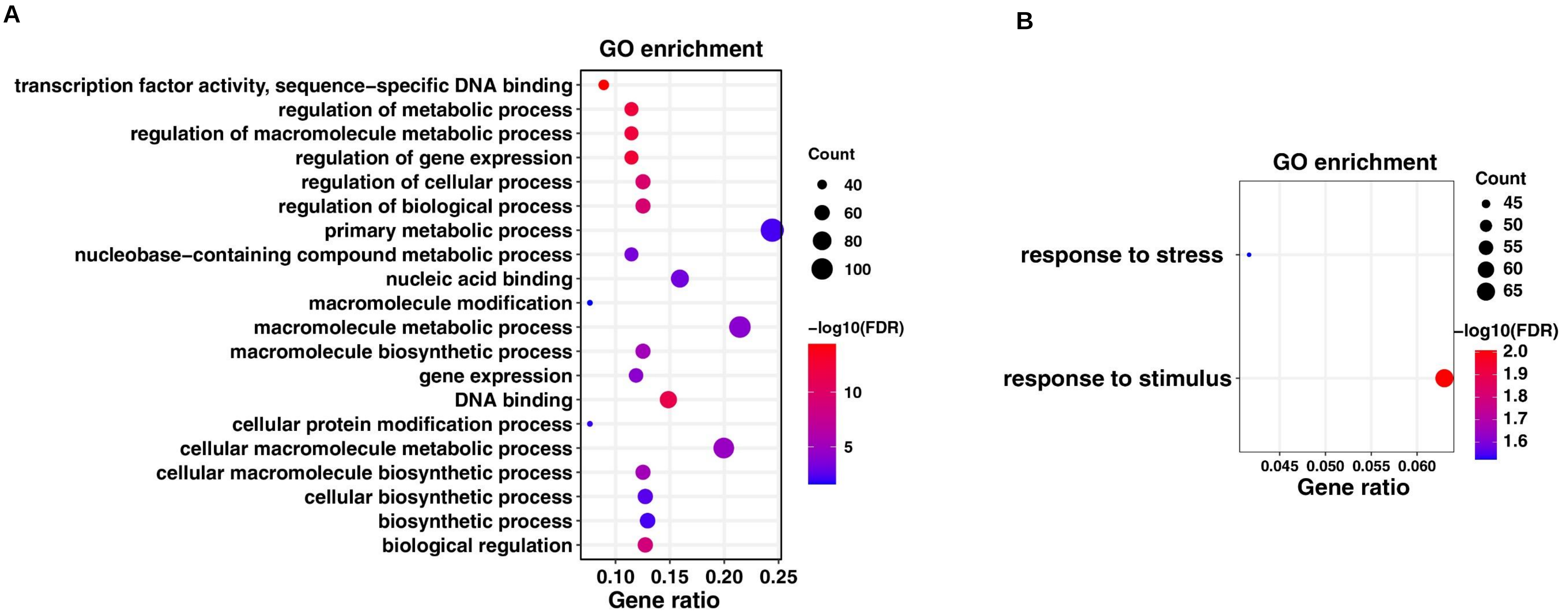

Additional file 1: Fig. S11 (A) GO term enrichment analysis of genes in the pink module. **(B)** GO term enrichment analysis of genes in the brown module.

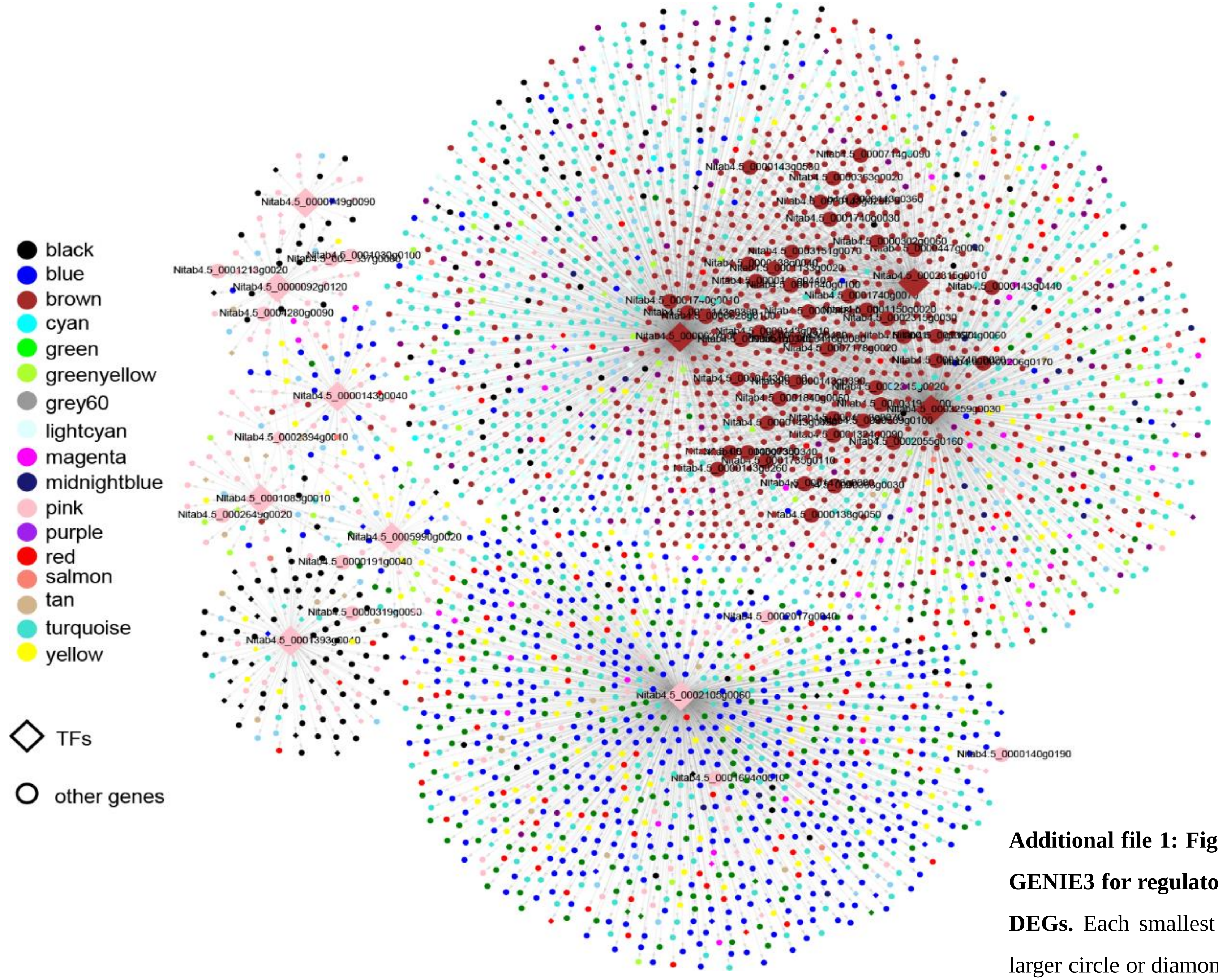

Additional file 1: Fig. S12 Genetic regulatory networks (GRNs) constructed by GENIE3 for regulatory relationship between 11 transcription factors and 3,308 DEGs. Each smallest circle or diamond represents the common DEGs, and each larger circle or diamond represents hub genes/TFs.

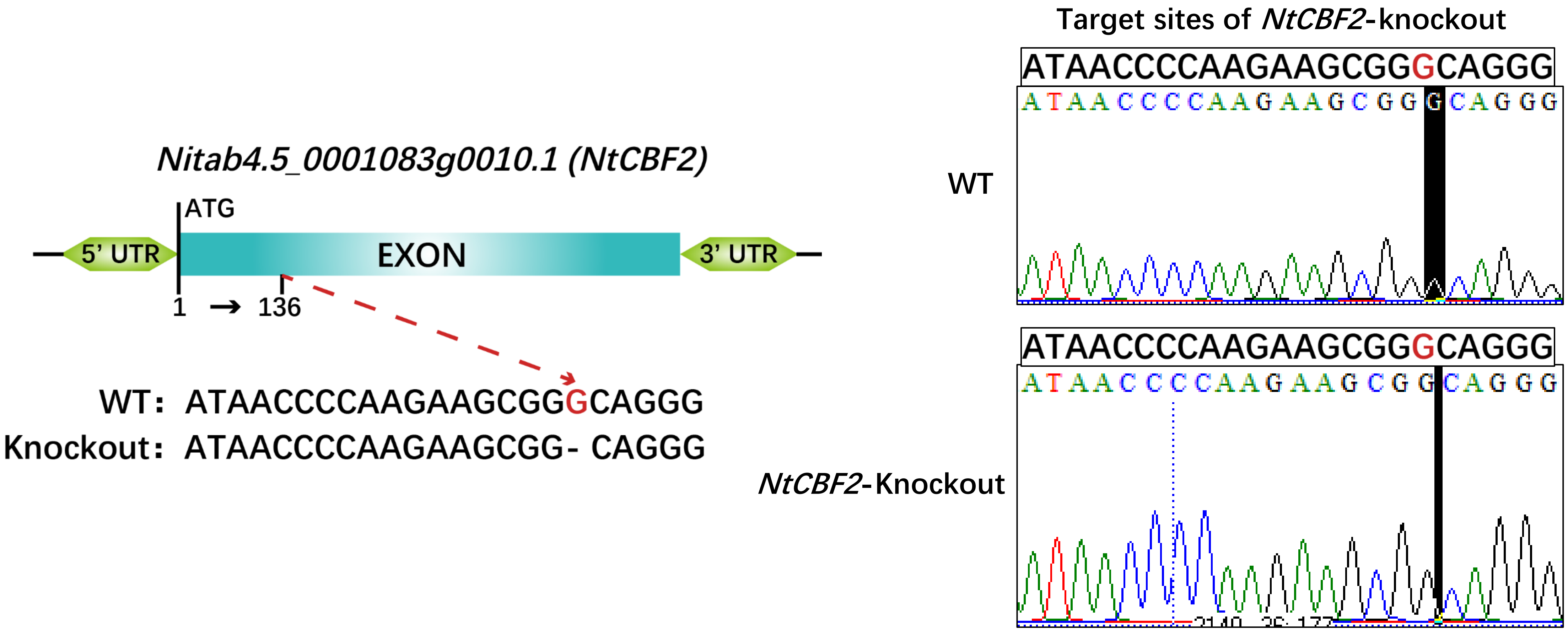

Additional file 1: Fig. S13 Sanger sequencing confirming the CRISPR-Cas9 related transgenic line with sequence mutation in the exon of *NtCBF2*.

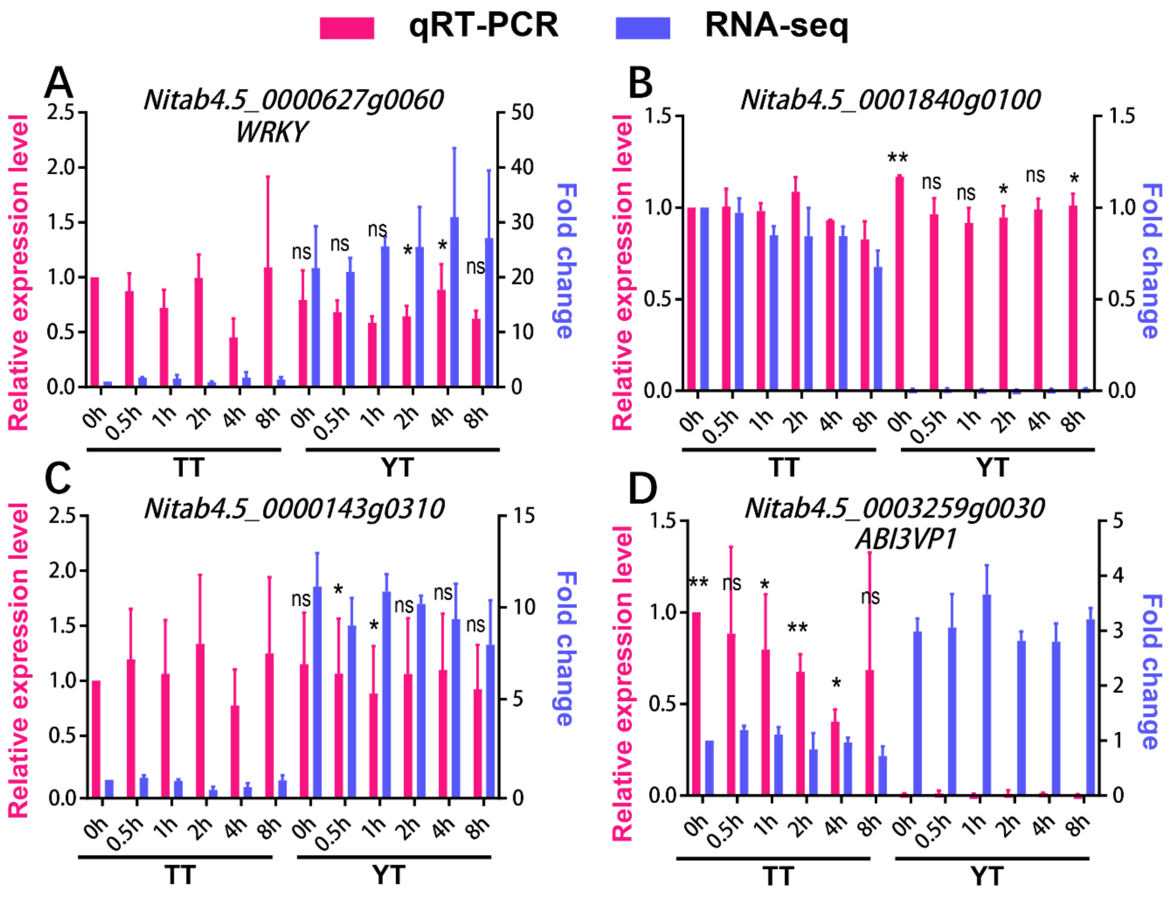

Additional file 1: Fig. S14 qRT-PCR validation of 4 key genes identified in the network. A dual y-axis plot illustrating in-parallel comparison between RNA-seq (expression fold change compared to 0 hours of TT, blue) and qRT-PCR (relative expression levels, red) of each individual gene examined. The right coordinate (y axis) with blue represents expression fold change of RNA-seq, the left coordinate (y axis) with red represents the relative expression levels of qRT-PCR. The transcript levels of each gene in each time point were normalized relative to the internal control of gene *NtActin7*. Relative expression levels of genes examined were calculated and expressed as $2^{-\Delta\Delta CT}$ relative to the expression levels of the corresponding genes in *NtActin7*, which were set as 1.0. The mean expression levels were calculated from three biological replicates. Error bars are standard deviations of three biological replicates. Significance test was determined using Student's t test. “*”: p<0.05, “**”: p<0.01, “***”: p<0.001.

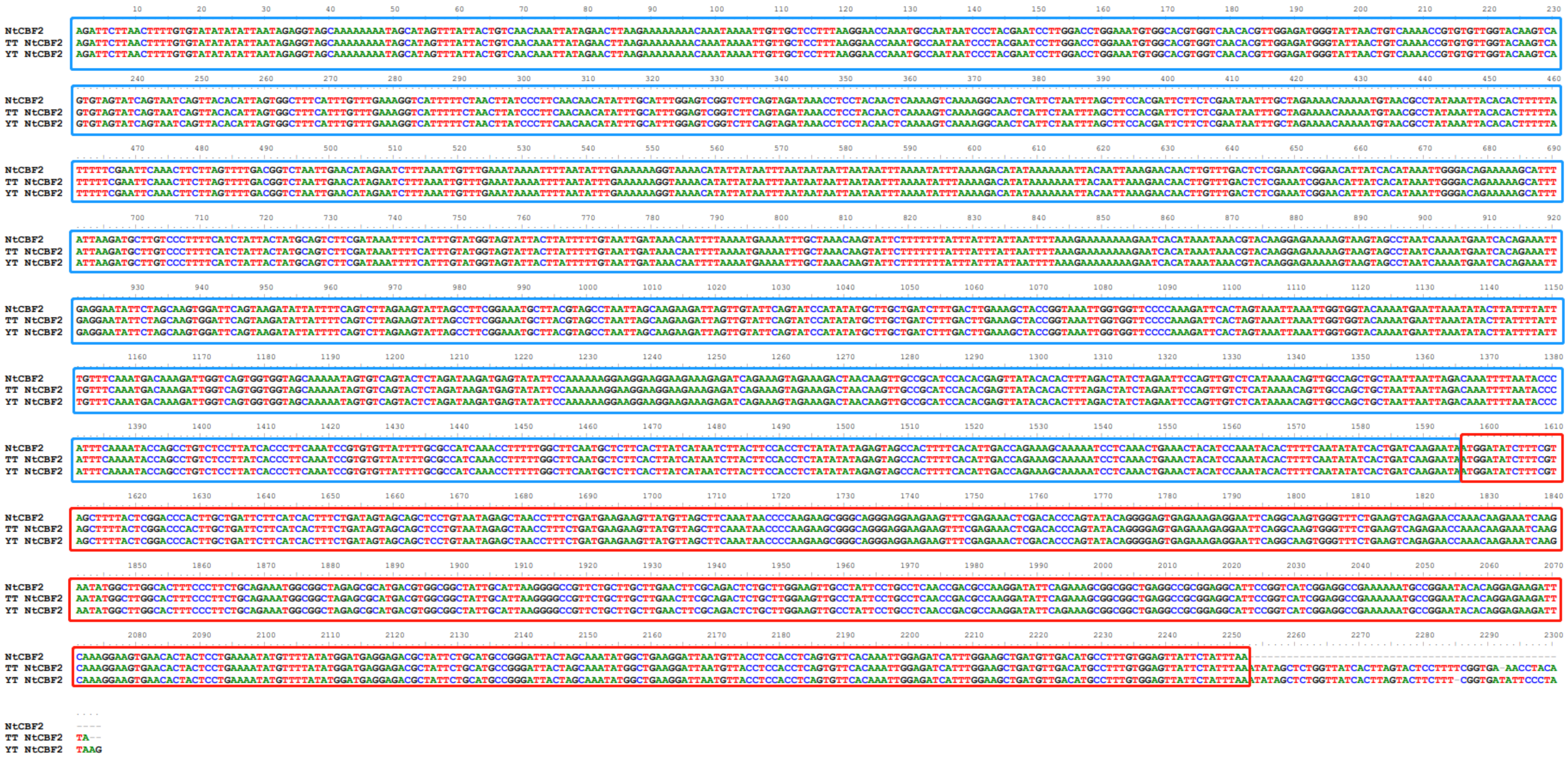

Additional file 1: Fig. S15 Comparisons of cDNA (red box) and promoter (1.5 kb upstream of ATG, blue box) sequences of *NtCBF2* between YT and TT. Red box marks CDS, blue box marks promoter regions.
